# Supplementary material for: Designing a norepinephrine optical tracer for imaging individual noradrenergic synapses and their activity in vivo
Source: Nat Commun. 2018 Jul 19;9:2838. doi: 10.1038/s41467-018-05075-x (PMC6053466; doi:10.1038/s41467-018-05075-x)
Supplement: Supplementary file 1 — Supplementary Information [file 41467_2018_5075_MOESM1_ESM.pdf]

SUPPORTING INFORMATION

Designing A Norepinephrine Optical Tracer for Imaging Individual Noradrenergic Synapses and Their Activity *in vivo*

Name	Structure	pKa
201	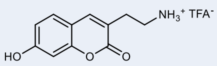	8.02
093	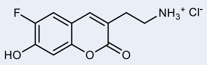	6.15
FFN202	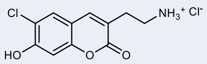	6.41
FFN270	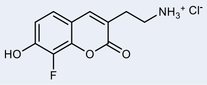	6.25
269	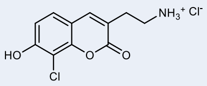	5.95
101	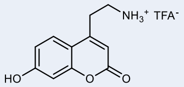	7.81
103	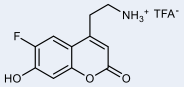	5.95
FFN102	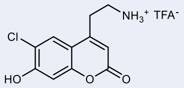	6.18

Supplementary Table 1. NE-FFN Candidates and pK_a's. List of FFN candidates tested for NET substrate activity. pKa was determined by measuring the change in absorbance of the red-shifted peak in PBS solutions of varying pH.

5-HT1A	5.7	Beta1	-17.3
5-HT1B	37.3	Beta2	-7.9
5-HT1D	-4	Beta3	-2.7
5-ht1e	11.5	D1	-2.5
5-HT2A	6.3	D2	-3.5
5-HT2B	3.5	D3	3.7
5-HT2C	2.5	D4	20.7
5-HT3	-8.5	D5	10.7
5-HT4	-2.8	DAT	34.2
5-ht5a	2.6	DOR	6.7
5-HT6	-4.4	GABAA	10.5
5-HT7	2.5	H1	5.6
A2B2	-3.4	H2	14.6
A2B4	5.5	H3	13.6
A3B2	-8.6	H4	2.8
A3B4	5.1	HERG binding	-4
A4B2	-3.6	KOR	7.3
A4B2	3.8	M1	5.6
A4B4	4.6	M2	-0.6
A7	-28.4	M3	-12.2
A7	6	M4	10.1
Alpha1A	-4.2	M5	1.5
Alpha1B	2.3	MOR	15.7
Alpha1D	-6.2	NET	14.3
Alpha2A	-2	SERT	-9
Alpha2B	-13.7	Sigma 1	-4.9
Alpha2C	-8.5	Sigma 2	2.2

Supplementary Table 2. Primary Screen of FFN270 binding at 54 CNS Receptors. FFN270 (10 μM) was tested at 54 CNS receptors in collaboration with the Psychoactive Drug Screening Program (PDSP). Data represent mean % inhibition (N = 4 determinations) for FFN270 tested at receptor subtypes. Values greater than 50% are considered significant.[1]

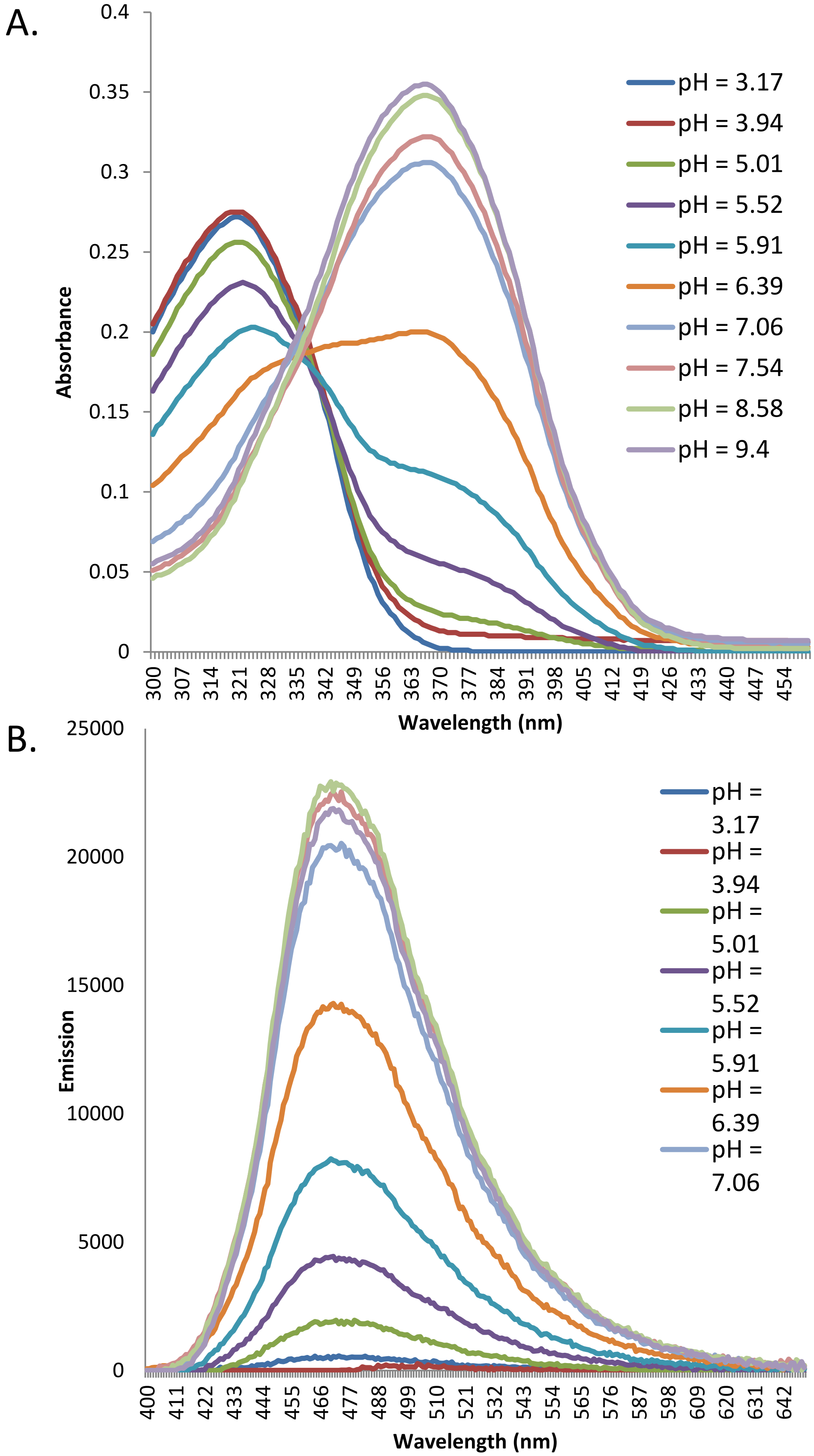

Supplementary Figure 1. FFN270 absorbance and emission spectra. A) Absorbance spectrum was obtained using 40 μM solutions of FFN270 in PBS buffer with pH values ranging from 3 to 10. B) Emission spectrum was obtained using 0.2 μM FFN270 solutions in the same buffers, excited at 365 nm wavelength.

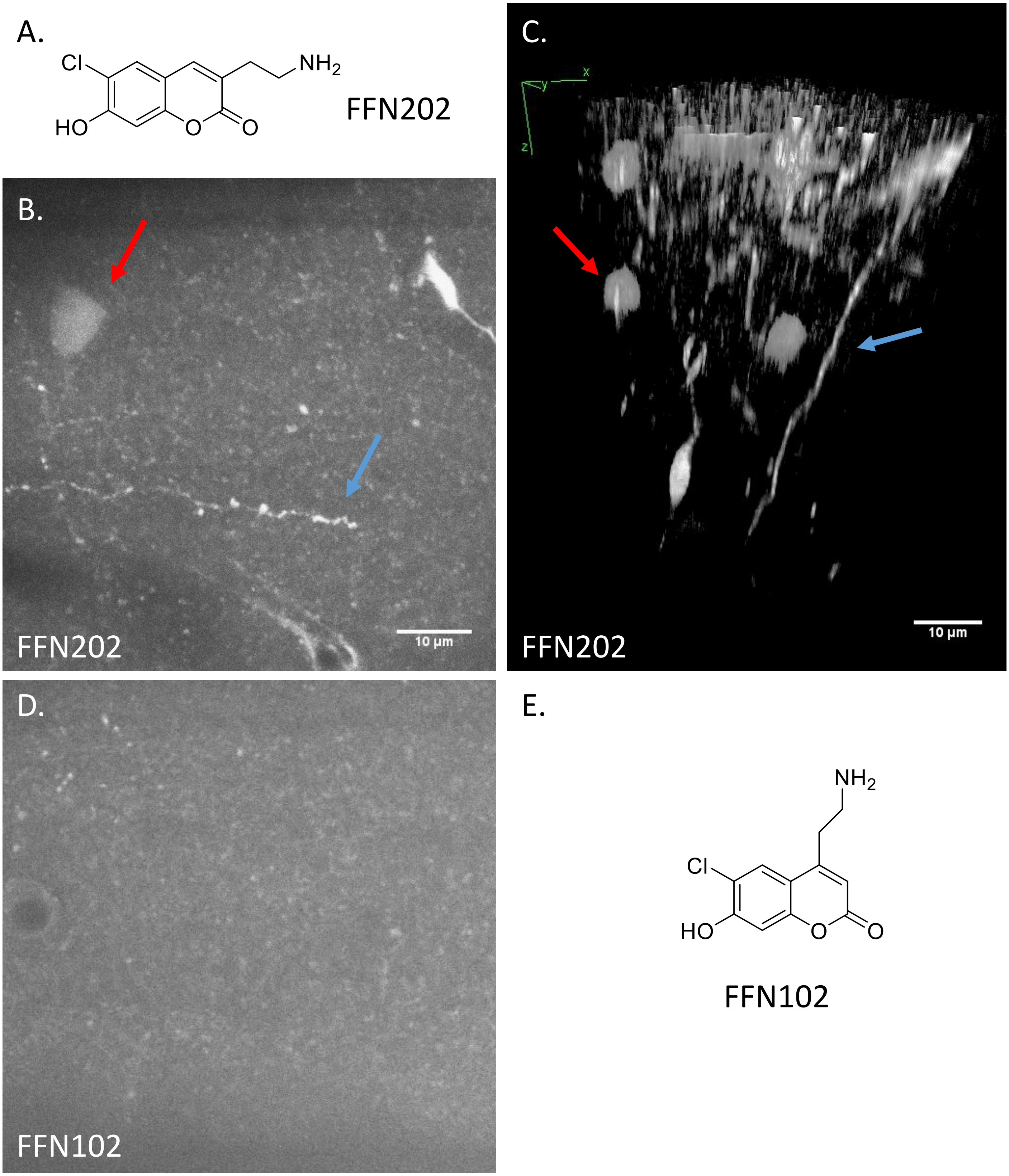

Supplementary Figure 2. FFN202 and FFN102 *in vivo*. Structures of FFN202 (A) and FFN102 (E), the first FFNs to be tested *in vivo* (Wenbio Gan, NYU). Two-photon fluorescence images of FFN202 (B) and FFN102 (D) taken in Layer 1 of the somatosensory cortex. Red arrow: cell body, Blue arrow: axonal structure. C) 3-D reconstruction of FFN202 labeling in the outermost 100 µm of somatosensory cortex. More axonal structures are observed traveling in the z-plane (blue arrow).

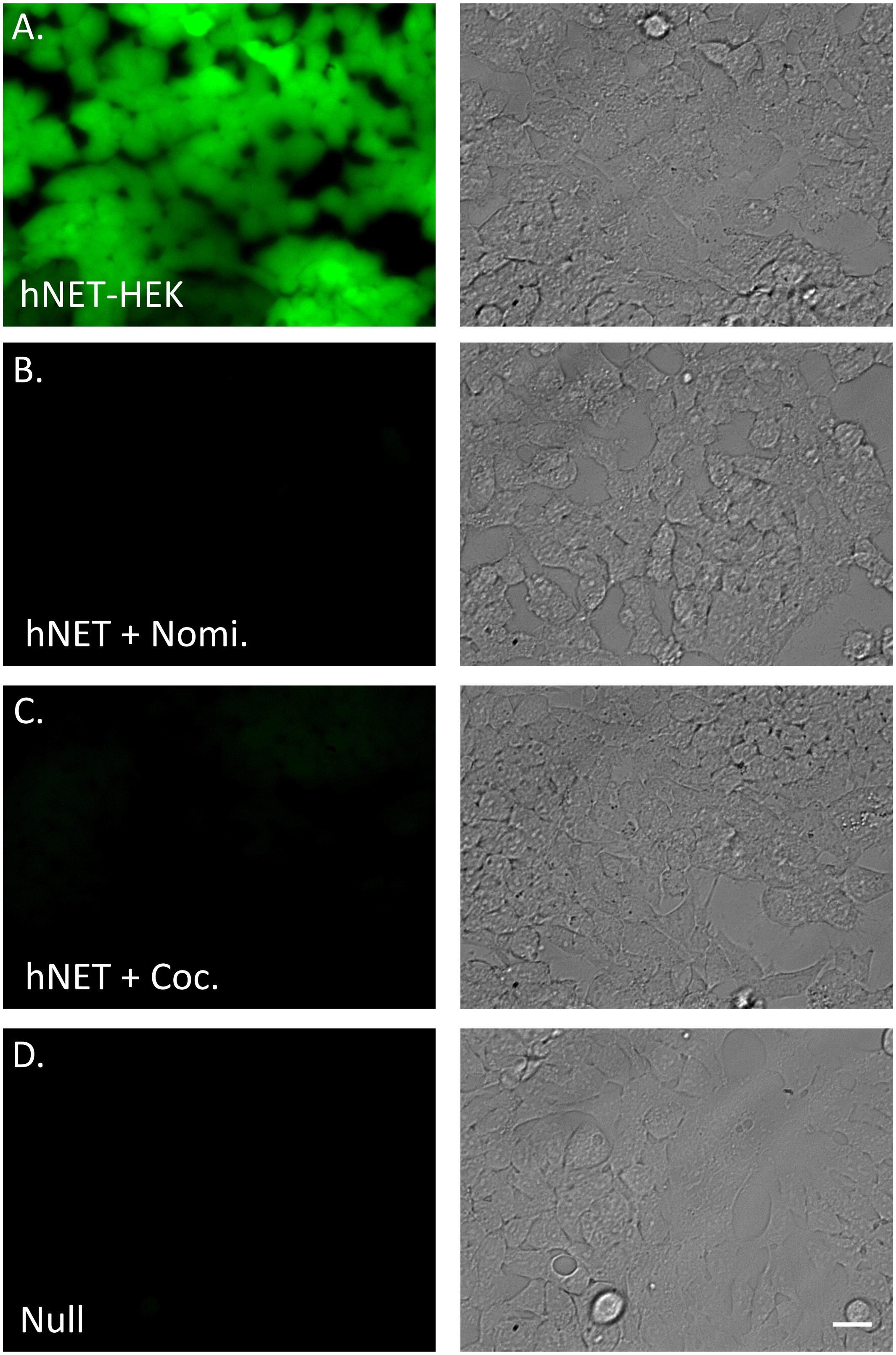

Supplementary Figure 3. FFN270 staining in hNET-HEK cells. Fluorescence (left) and corresponding bright-field images (right) of hNET-transfected HEK cells treated with 20 µM FFN270 in the absence (A) or presence of nomifensine (2 µM, B) or cocaine (1 µM, C). D) Null-transfected HEK cells treated with 20 µM FFN270. Scale Bar: 20 µm.

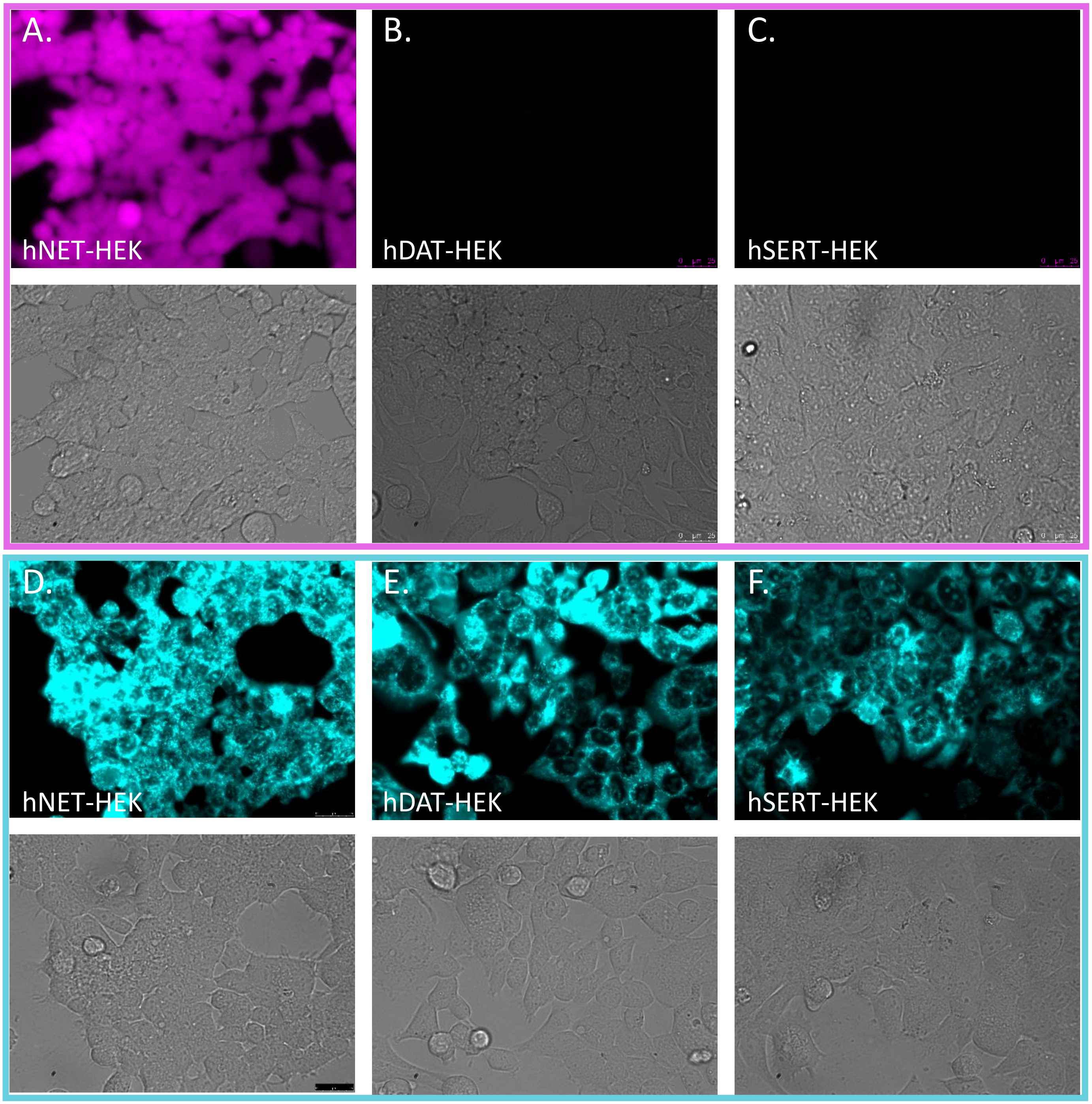

Supplementary Figure 4. FFN270 uptake through hNET, hDAT, and hSERT. Fluorescence and bright-field images of HEK cells transfected with hNET, hDAT, or hSERT. A-C) Cells are treated with 20 µM FFN270. D-F) As a positive control, the same cell lines are treated with 1 µM APP+, a known fluorescent substrate of all three transporters, to control for transporter expression. Scale Bar: 20 µm.

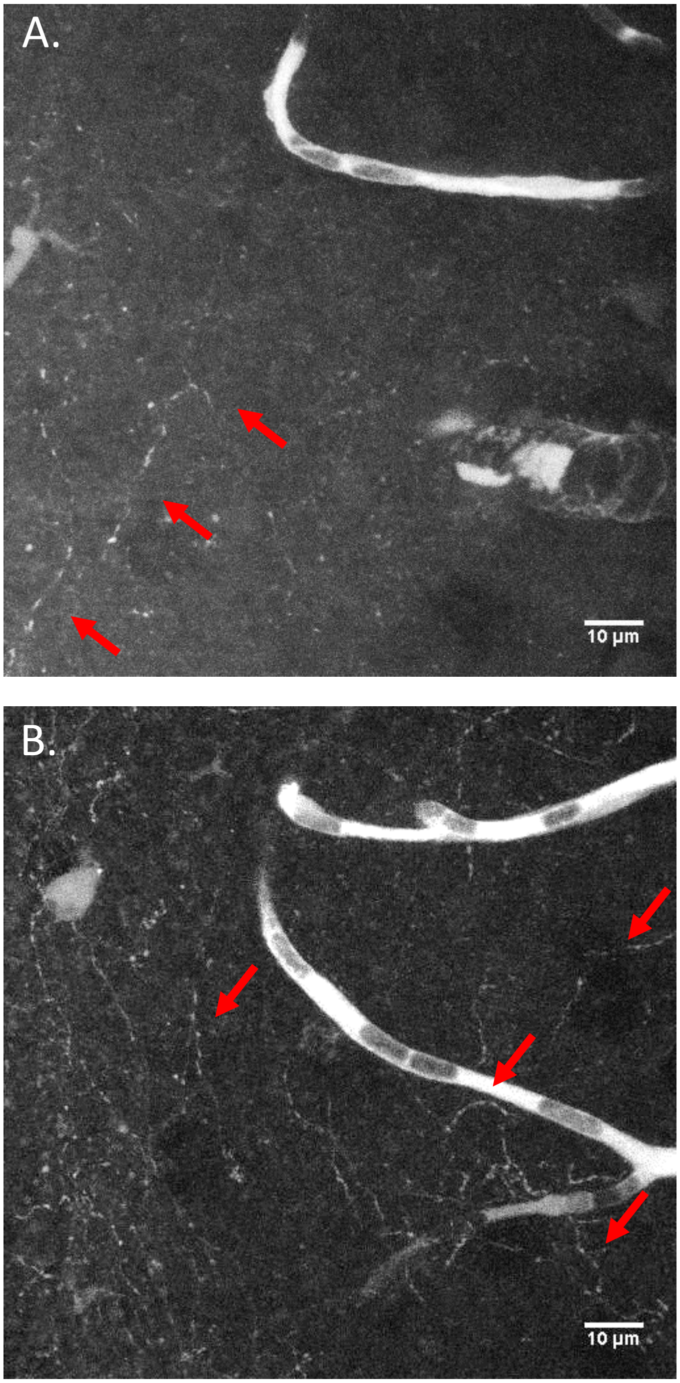

Supplementary Figure 5. FFN202 and 093 staining Layer 1 of the barrel cortex in acute murine brain slice. Representative images of FFN202 (A) and **093** (B) in Layer 1 of the barrel cortex following a 30 min incubation (10 µM). Highlighted with red arrows are labeled axon structures. Scale Bar: 10 µm.

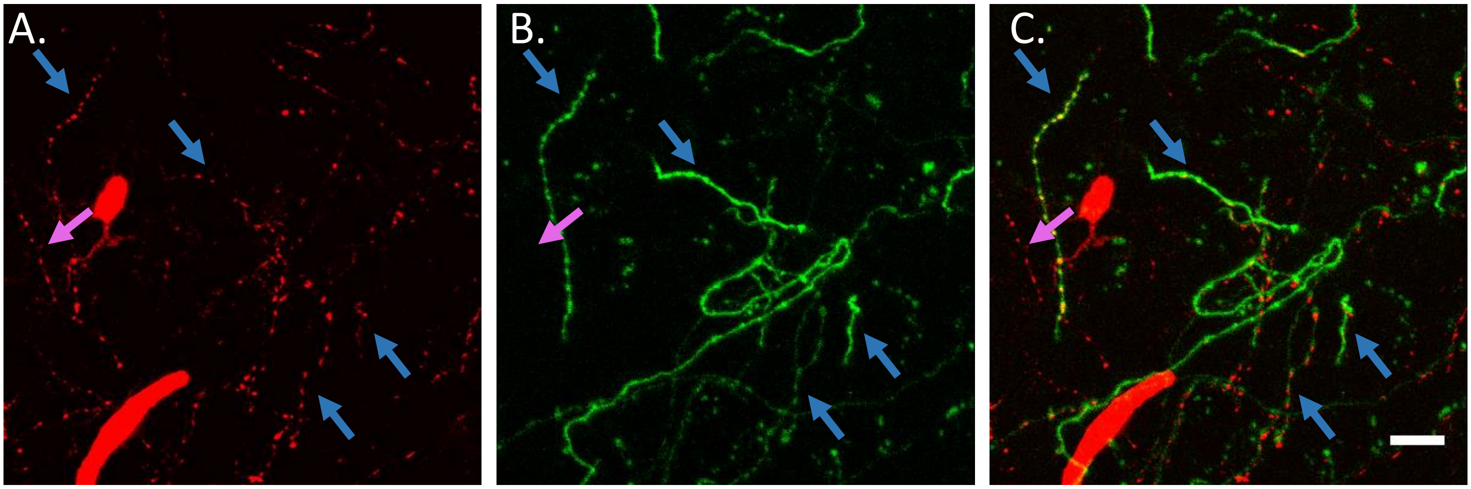

Supplementary Figure 6. ChR2-YFP expression in NE-neurons and colocalization with FFN270. ChR2-YFP was expressed in noradrenergic neurons as described in the Methods, and then the YFP was imaged in the barrel cortex as a positive marker for noradrenergic projections (B). A) FFN270 staining in the same area. C) colocalization between the two channels can be observed in some axons (highlighted with blue arrow) and not in others (highlighted with a pink arrow). Scale bar: 10 µm.

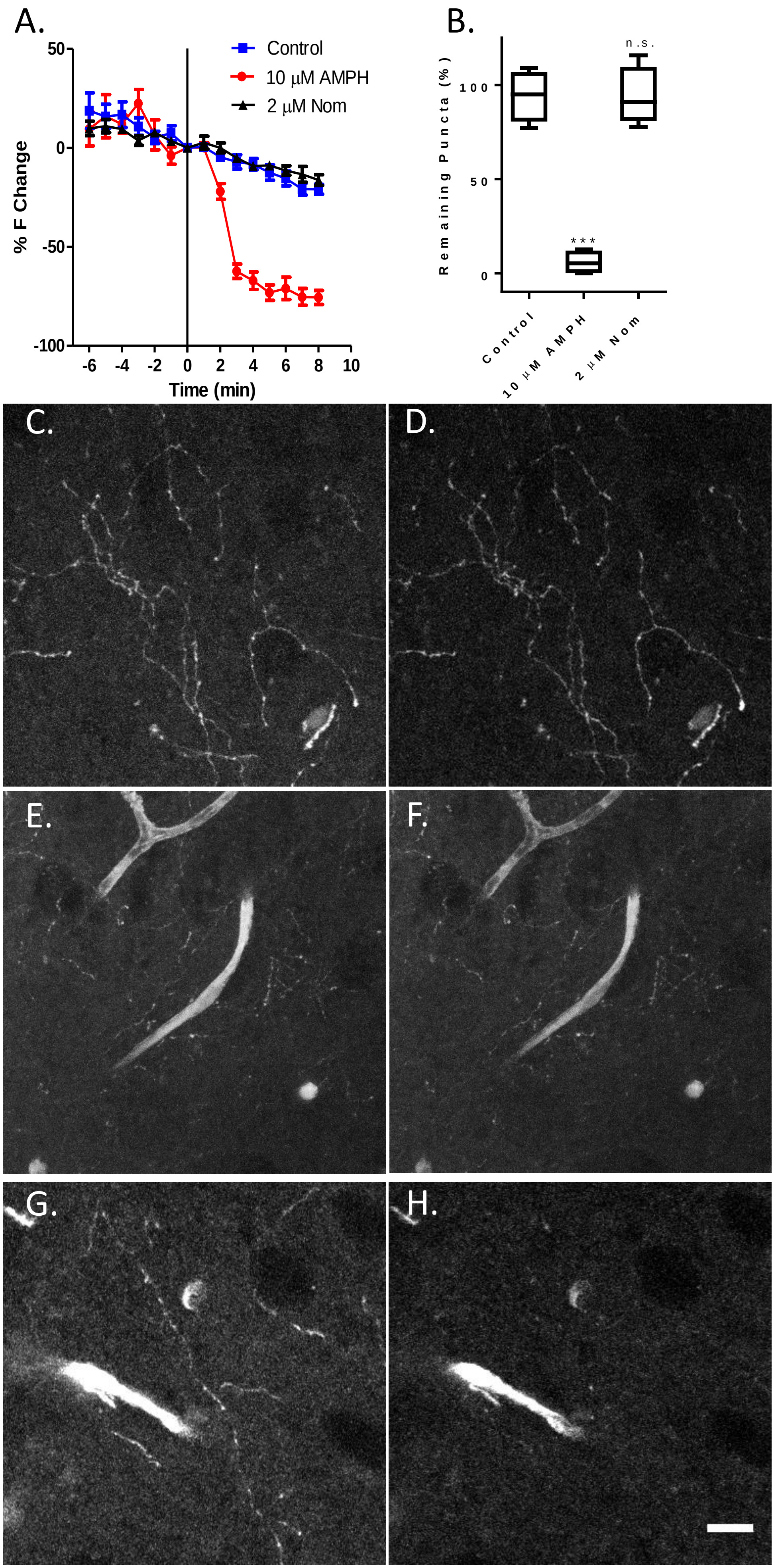

Supplementary Figure 7. Amphetamine-induced FFN270 release in acute brain slice. A) Acute slices were loaded with FFN270 and then imaged every 1 min. The average change in fluorescence of FFN270 puncta was measured during the course of an amphetamine (AMPH, 10 µM), nomifensine (Nom., 2 µM), or ACSF perfusion (starting at t = 0). B) The number of selected FFN270 puncta before and 5 min after AMPH was significantly different compared to controls (P < 0.0001, one-way ANOVA Dunnett post-hoc) while Nom. perfusion showed no difference (P = 0.95). Data from each condition is collected from 2-3 slices per animal from 3 animals, and presented with min to max whiskers. Representative FFN270 images before and 5 min after ACSF (C-D), Nom. (E-F), or AMPH (G-H) perfusion. Scale bar: 10 µm.

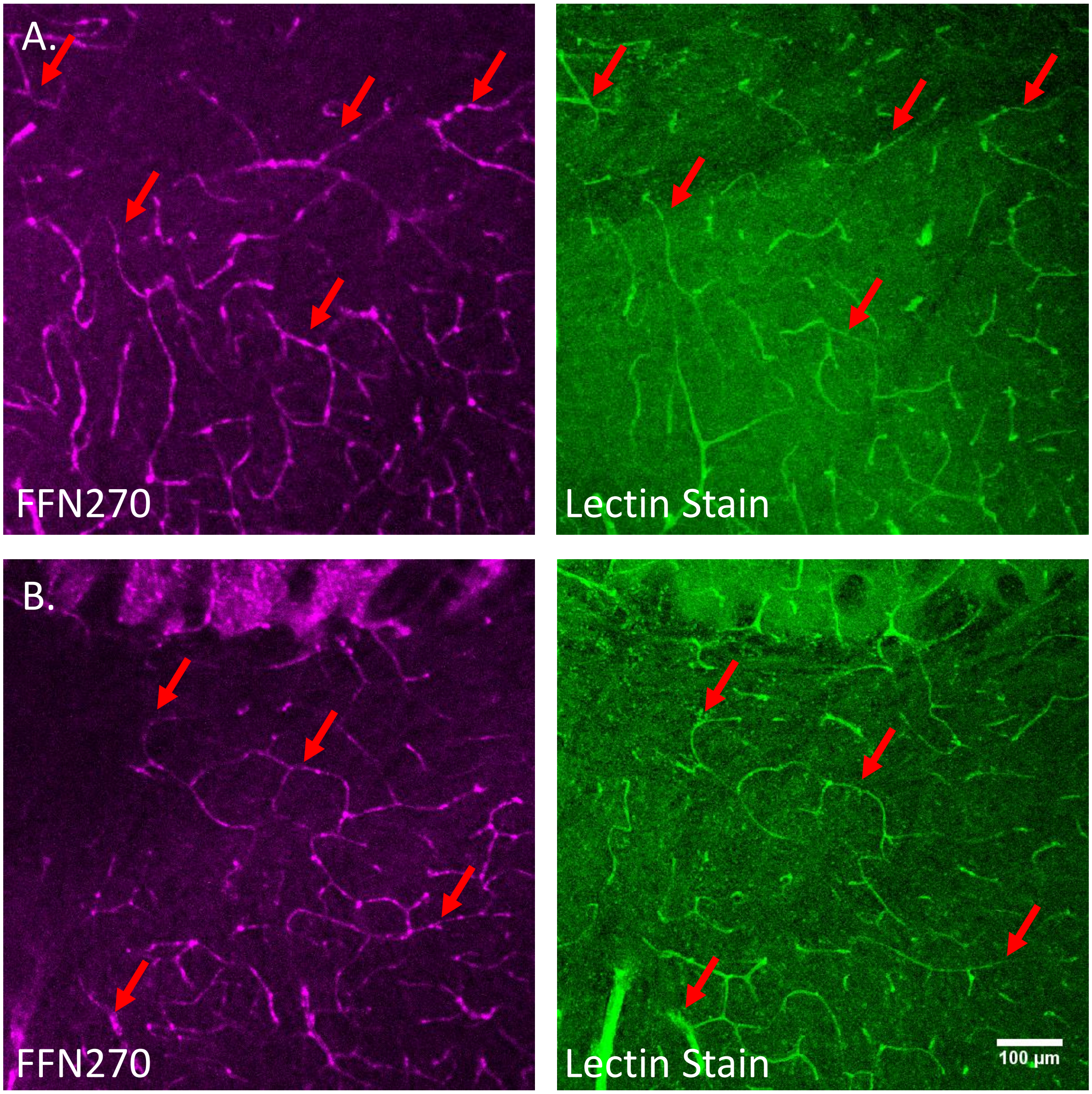

Supplementary Figure 8. Colocalization of FFN270 with blood vessels. A-B) Two representative pairs of low zoom images highlighting colocalization between FFN270 (magenta) and lectin stained vasculature (green) across Layers 2-6 of the barrel cortex. Note that there is not perfect overlap between channels as the FFN270 images were collected in healthy acute brain slices, and the lectin stain was performed on those same slices post-fixation. Some clearer examples have been highlighted by red arrows. Scale bar: 100 µm.

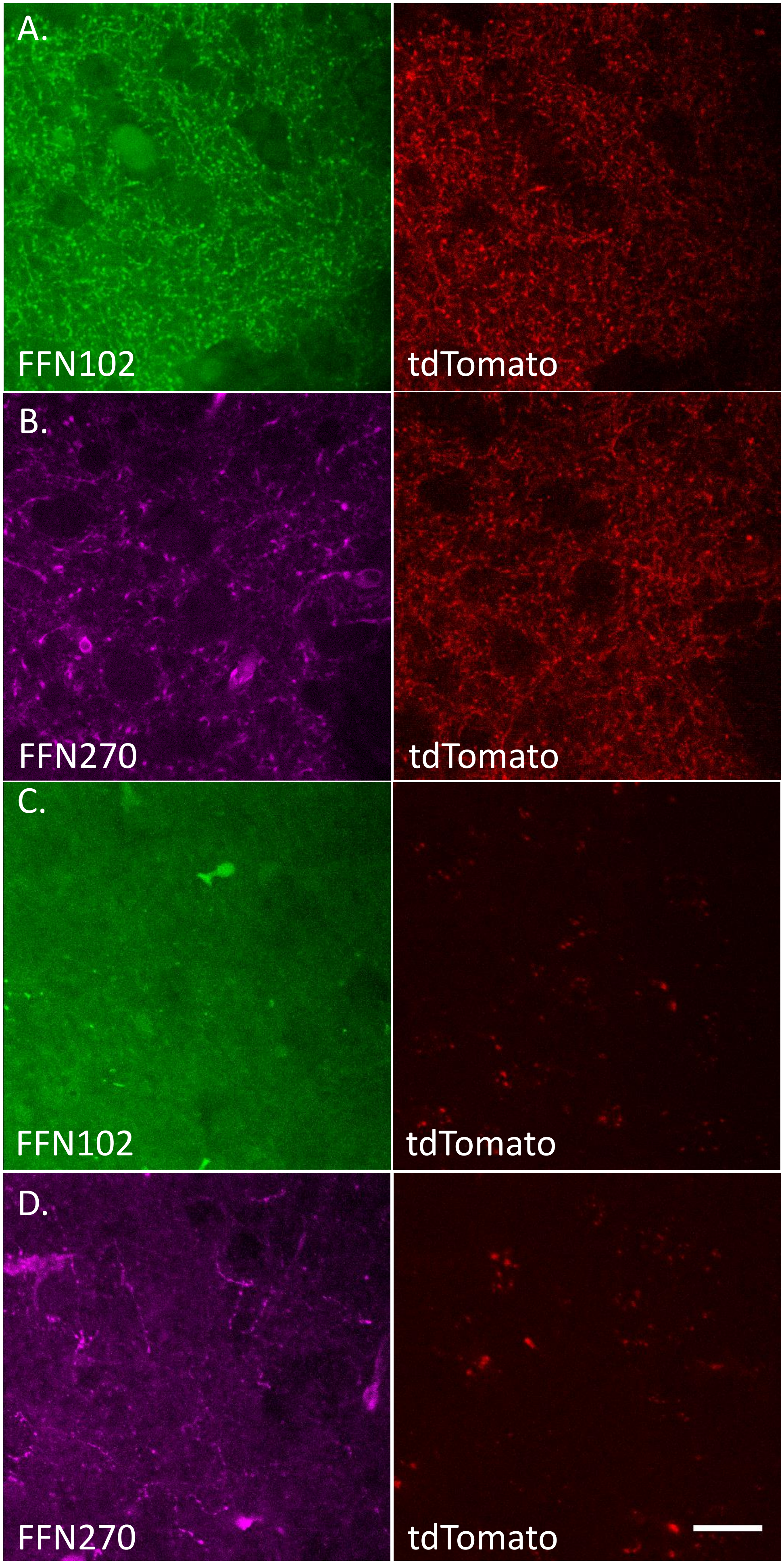

Supplementary Figure 9. Comparison of FFN102 and FFN270 loading in dorsal striatum and barrel cortex. A-B) Acute slices containing the dorsal striatum (DS) were loaded with a high incubation dose (50 µM) of either FFN102 (green) or FFN270 (magenta), and colocalization with the tdTomato reporter (red) in DAT-positive neurons was determined (see Methods for details). We observed much stronger FFN102 signal in the DS compared to FFN270, even at this elevated dose. C-D) This comparison was repeated in the barrel cortex, where we observed almost no dopamine axons (small scattered spots are likely autofluorescence). In this brain region, we observed insignificant FFN102 uptake compared to FFN270, even with 50 µM incubation. Scale bar: 20 µm.

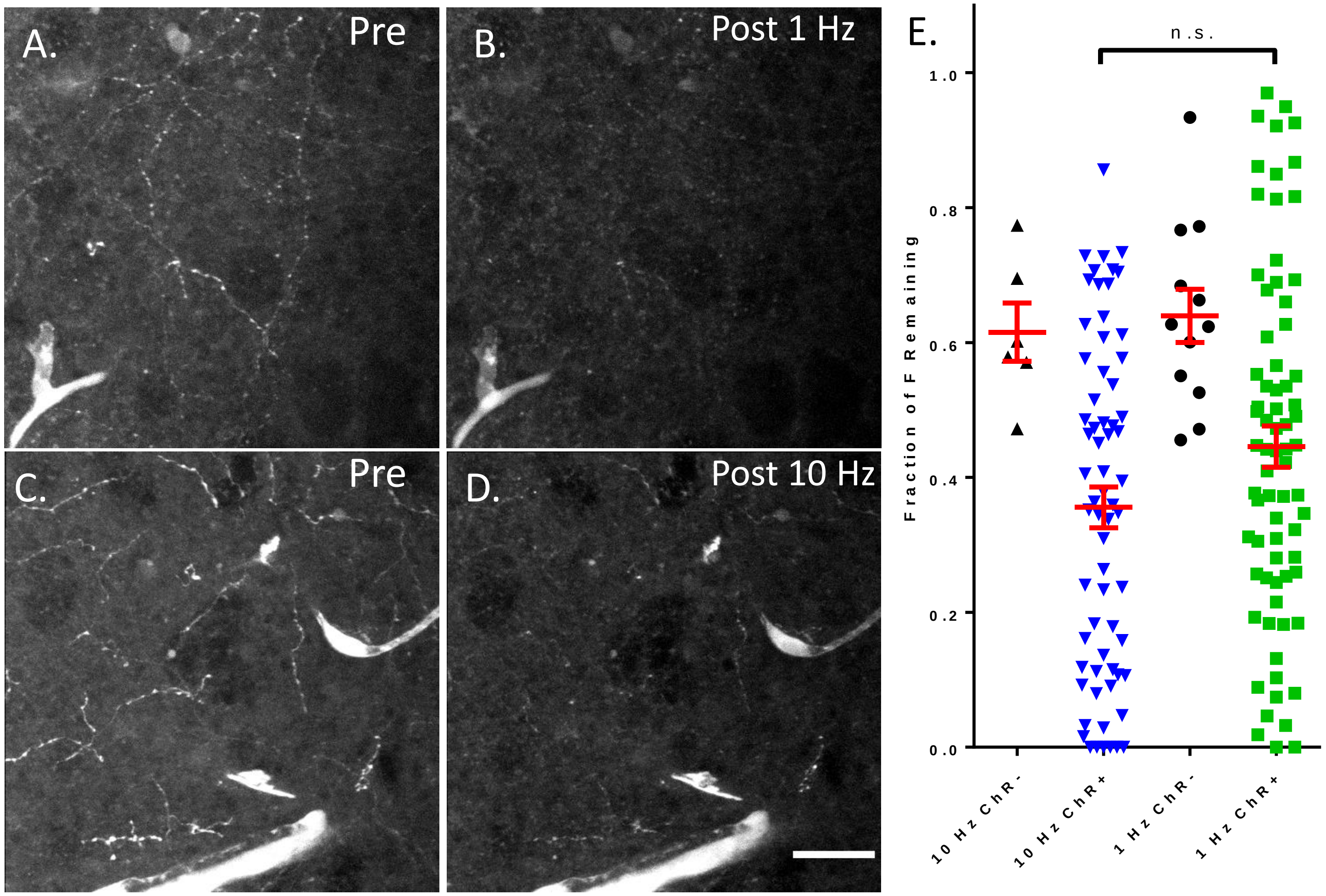

Supplementary Figure 10. Effect of optogenetic stimulation frequency on FFN270 release. Acute murine brain slices containing Layer 1-3 of the barrel cortex were collected from TH-Cre/Ai32 animals expressing ChR2-EYFP in monoaminergic neurons. A-B) Representative images of FFN270 before (A) and after (B) 4 min of 1 Hz 470 nm light stimulation (5 ms duration, 240 pulses). C-D) Representative images of FFN270 before (A) and after (B) 4 min of 10 Hz 470 nm light stimulation (5 ms duration, 2,400 pulses). E) Change in fluorescence of individual axons was quantified and then grouped depending on colocalization with the EYFP reporter. Average release ± SEM following 10 Hz (65.4 ± 3.0%) and 1 Hz (55.5 ± 3.0%) were not significantly different each other (Mann-Whitney, *P* = 0.07, *n* = 2 slices per condition per animal, 4 animals) and comparable to previous observations (Fig. 6). Scale Bar: 20 μm.

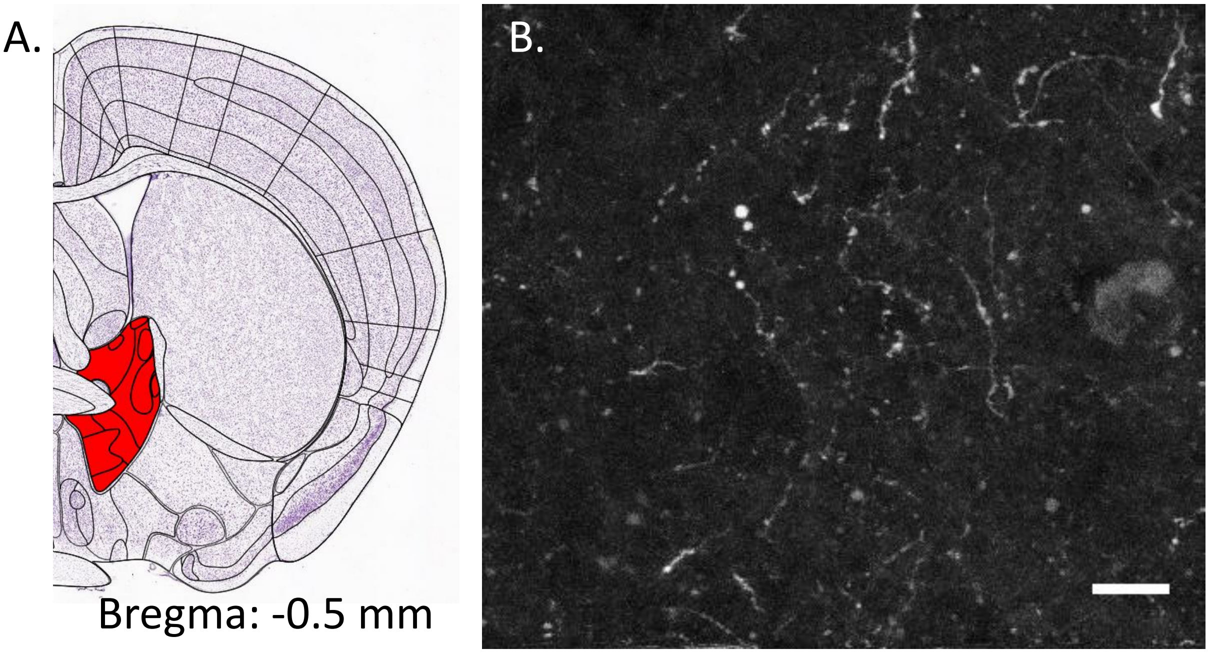

Supplementary Figure 11. FFN270 Loading in Bed Nuclei of the Stria Terminalis. A) Atlas image highlighting in red the location of the bed nuclei of the stria terminalis (BST) in the mouse brain (Bregma: -0.5 mm, Allen Institute).[2] B) FFN270 (10 µM) labeling pattern in the BST following 30 min incubation. Scale Bar: 10 µm.

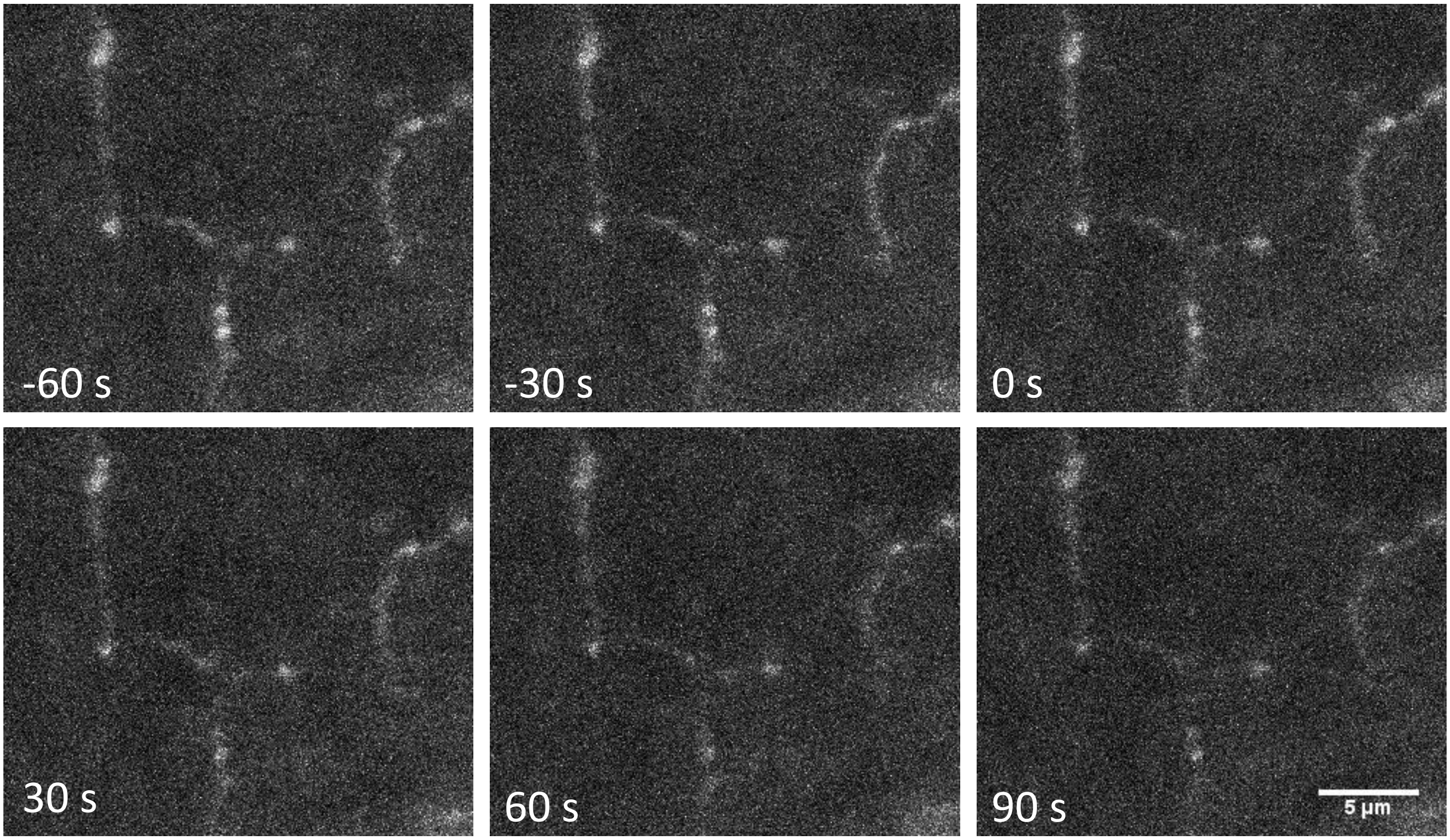

Supplementary Figure 12. FFN270 Electrical Stimulation with Cadmium ions. A representative set of images highlighting the change in FFN270 fluorescence signal over the course of a 10 Hz electrical stimulus while blocking calcium channels with cadmium chloride (200 μM). See Figure 5 as a comparison with control FFN270 fluorescent changes. Scale Bar: 5 μm.

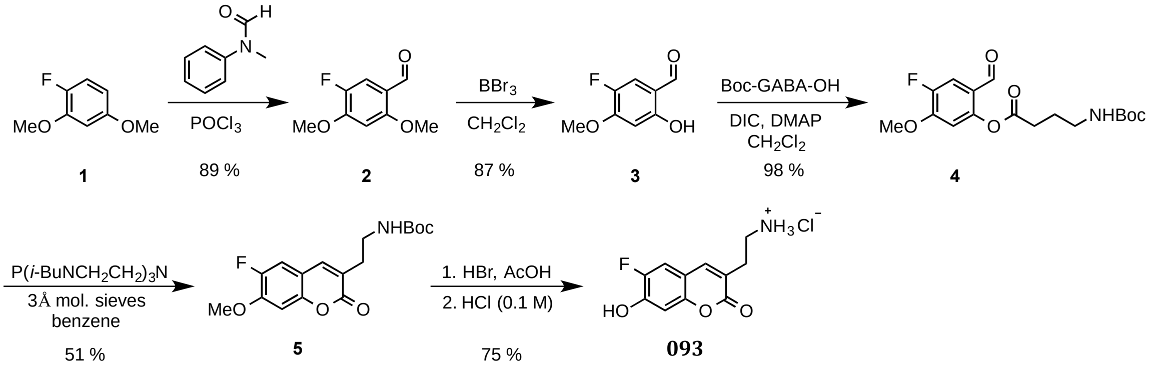

Supplementary Figure 13. Synthesis of 093. Formylation of flourodimethoxybenzene **1** was carried out using a modified Vilsmeier-Haack reagent. After a selective deprotection of methoxy group in position 2 using boron tribromide, the free hydroxyl group of **3** was acylated with Boc-protected GABA in DIC mediated/DMAP catalyzed process. Cyclization of ester-aldehyde **4** in the presence of a catalytic amount of phosphaza-superbase and molecular sieves as a water scavenger yielded the coumarin **5**. Final deprotection of both Boc and methoxy group was achieved by heating the protected coumarin **5** with mixture of hydrobromic and acetic acid. HPLC purification and anion exchange yielded the final probe **093** as an HCl salt.

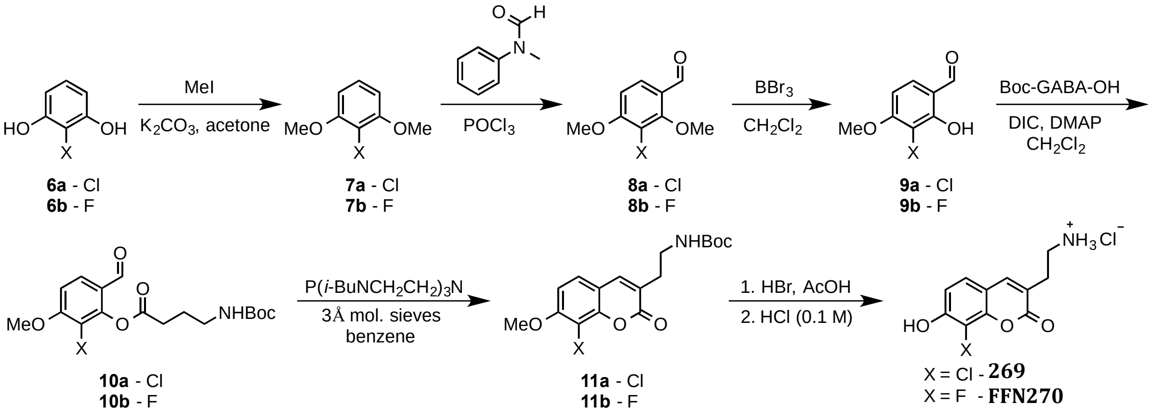

Supplementary Figure 14. Synthesis of 269 and FFN270. Commercially available 2-haloresorcinols **6** were protected by methylation. 2-Halodimethoxybenzenes **7** were converted to final coumarins **269** and **FFN270** in similar manner as **093** (Supplementary Figure 12).

Supplementary Methods. Synthesis and Characterization of the Probes. Unless otherwise noted, all chemicals were purchased from commercial companies and used without further purification. Nuclear Magnetic Resonance spectra were recorded on Bruker 400 and 500 MHz Fourier transform NMR spectrometers. Proton chemical shifts δ are expressed in parts per million (ppm) and are referenced to residual proton in the NMR solvent ($CDCl_3$, δ = 7.26 ppm; CD_3OD, δ = 3.31 ppm; $(CD_3)_2SO$, δ = 2.50 ppm; $(CD_3)_2CO$, δ = 2.05 ppm; D_2O, δ = 4.79 ppm). Data for ^1H NMR and ^{19}F NMR are reported as follows: chemical shift, integration, multiplicity (s = singlet, d = doublet, t = triplet, m = multiplet, br = broad peak, app = apparent), and coupling constant in Hz. Carbon chemical shifts are referenced to the carbon resonance of the NMR solvent ($CDCl_3$, δ = 77.2 ppm; CD_3OD, δ = 49.0 ppm; $(CD_3)_2SO$, δ = 39.5 ppm; $(CD_3)_2CO$, δ = 29.8 ppm). ^{13}C NMR spectra were recorded without ^{19}F decoupling and therefore J_{CF} splitting of ^{13}C signals was observed for fluorinated compounds. Low-resolution mass spectra were recorded on a JEOL LCmate instrument in $APCI^+$ ionization mode (Note: Mass spectra of Boc-protected compounds are reported for carbocations corresponding to loss of Boc fragment). High-resolution mass spectra (HR-MS) were recorded on a JOEL HX110 mass spectrometer in FAB ionization mode.

Preparative HPLC was performed on Waters 600 Controller equipped with a Vydac C_{18} Protein & Peptide column (mobile phase – gradient of solvents A and B, where A = deionized water containing 0.1% (v/v) formic acid (FA); B = HPLC grade methanol containing 0.1% (v/v) formic acid (FA)), Waters 2487 Dual Wavelength Absorbance Detector (λ = 254 nm) and Waters 2767 Sample Manager. Analytical HPLC was performed on the same instrument equipped with a Phenomenex reverse phase column (Prodigy 5 micron ODS3 100A 250 x 4.6 mm) using isocratic methanol:water (30:70) mobile phase. All probes were purified by preparative HPLC in the final stage and converted to a hydrochloride salt. The hydrochloride salts of final fluorophores were prepared by treating the formate salts with 4-6 molar excess of HCl (0.1 M) followed by concentration and lyophilization. The HCl salts were obtained as white powder.

The synthesis of FFN102, **103**, **201** and FFN202 were reported previously.[3]

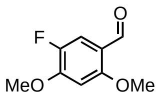

2

5-Fluoro-2,4-dimethoxybenzaldehyde (**2**)[4]

$POCl_3$ (0.60 mL, 6.40 mmol) was dropwise added to *N*-methylformanilide (0.79 mL, 6.40 mmol) and the resulting mixture was stirred at room temperature for 30 min. 2,4-Dimethoxyflurobenzene **1** (0.84 mL, 6.40 mmol) was then added and the mixture was heated to 35 °C for 3 h. After an overnight stirring at room temperature the reaction mixture was poured into ice-water. The white precipitate was collected by filtration and crystallized from cyclohexane affording the product as a white crystals (1.05 g, 89%). **^{1}H NMR (400 MHz, Acetone-*d*$_6$)** δ 10.25 (d, *J* = 3.3 Hz, 1H), 7.41 (d, *J* = 11.3 Hz, 1H), 6.95 (d, *J* = 6.7 Hz, 1H), 4.03 (s, 3H), 4.01 (s, 3H).

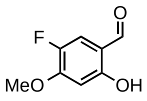

3

5-Fluoro-2-hydroxy-4-methoxybenzaldehyde (**3**)

BBr_3 (1.0 M solution in CH_2Cl_2, 0.9 mL, 0.9 mmol) was slowly added to an ice-cold solution of **2** (125 mg, 0.68 mmol) in dry CH_2Cl_2 (8 mL) under argon protection. The reaction mixture was then stirred at room temperature for 15 h, diluted with water and extracted with EtOAc (3x15 ml). The combined organic layers were dried over $MgSO_4$ and concentrated. The crude product was purified by column chromatography (hexane:CH_2Cl_2 – 1:2). The product **3** was obtained as a white solid (105 mg, 87%). **^{1}H NMR (400 MHz, Acetone-*d*$_6$)** δ 9.81 (d, *J* = 0.3 Hz, 1H), 7.52 (d, *J* = 10.9 Hz, 1H), 6.68 (d, *J* = 7.0 Hz, 1H), 4.00 (s, 3H).[5]

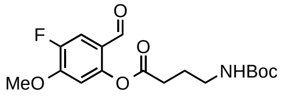

4

4-Fluoro-2-formyl-5-methoxyphenyl 4-((*tert*-butoxycarbonyl) amino)butanoate (**4**)

To a solution of compound **3** (130 mg, 0.76 mmol), Boc-GABA-OH (185 mg g, 0.92 mmol) and 4-(dimethylamino)pyridine (10 mg) in CH_2Cl_2 (12 mL) was added *N,N'*-diisopropylcarbodiimide (0.16 mL, 1.0 mmol). The reaction mixture was stirred at room temparature for 1.5 h, washed with H_2O, brine and dried over $MgSO_4$. The crude product was purified by column chromatography (CH_2Cl_2 + 3% acetone) affording the compound **4** as a white solid (265 mg, 98%). **^{1}H NMR (400 MHz, Acetone-*d*$_6$)** δ 10.00 (d, *J* = 1.6 Hz, 1H), 7.59 (d, *J* = 11.1 Hz, 1H), 7.17 (d, *J* = 7.1 Hz, 1H), 6.09 (br s, 1H), 4.00 (s, 3H), 3.22 (q, *J* = 6.5 Hz, 2H), 2.76 (t, *J* = 7.3 Hz, 2H), 1.91 (p, *J* = 6.9 Hz, 2H), 1.42 (s, 9H); **^{19}F NMR (376 MHz, Acetone-*d*$_6$)** δ -137.78 (ddd, *J* =

10.4, 7.1, 2.2 Hz); **^{13}C NMR (101 MHz, Acetone-*d*$_6$)** δ 187.5, 172.3, 157.0, 154.3, 154.2, 152.0, 150.8, 149.6, 122.3, 115.9, 115.7, 109.9, 78.6, 57.3, 40.0, 31.5, 28.7, 26.0. **HR-MS** calcd. for $C_{17}H_{22}FNNaO_6$ $[M+Na]^+$ 378.1329, found 378.1332.

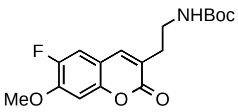

5

***tert*-Butyl (2-(6-fluoro-7-methoxy-2-oxo-2*H*-chromen-3-yl)ethyl)carbamate** (**5**)

To a suspension of compound **4** (390 mg, 1.1 mmol) and activated 3Å molecular sieves (powder, 1.5 g) in dry benzene (10.0 mL) was added 2,8,9-triisopropyl-2,5,8,9-tetraaza-1-phospha- bicyclo[3.3.3]undecane (170 mg, 0.51 mmol) in dry benzene (5.0 mL) via syringe at 50 °C. After being stirred at 50 °C under argon for 16 h, the reaction mixture was cooled to RT, diluted with CH_2Cl_2 (20 mL), filtered through celite and concentrated. Purification by column chromatography (hexane:ethyl acetate – 5:2) yielded compound **5** as a white solid (205 mg; 55%). **^{1}H NMR (400 MHz, Acetone-*d*$_6$)** δ 7.63 (s, 1H), 7.39 (d, *J* = 11.0 Hz, 1H), 7.09 (d, *J* = 7.2 Hz, 1H), 6.07 (br s, 1H), 4.00 (s, 3H), 3.36 (q, *J* = 6.3 Hz, 2H), 2.66 (t, *J* = 6.4 Hz, 2H), 1.31 (s, 9H); **^{19}F NMR (376 MHz, Acetone-*d*$_6$)** δ -140.10 (dd, *J* = 10.9, 7.3 Hz); **^{13}C NMR (101 MHz, Acetone-*d*$_6$)** δ 161.7, 156.7, 151.7, 150.9, 148.5, 140.0, 125.6, 113.8, 113.6, 113.1, 113.0, 102.2, 78.5, 57.1, 39.3, 32.8, 28.5; **HR-MS** calcd. for $C_{17}H_{20}FNNaO_5$ $[M+Na]^+$ 360.1223, found 360.1227.

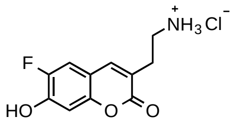

093

3-(2-Aminoethyl)-6-fluoro-7-hydroxy-2*H*-chromen-2-one hydrochloride salt (**093**)

A solution of compound **5** (65 mg, 0.185 mmol) in AcOH (1.5 mL) and HBr (1.5 mL) was heated in closed vial to 110 °C for 38 h. The reaction mixture was then concentrated and the crude product was purified by reverse phase HPLC using a linear gradient - water (A), MeOH (B) (25% B → 95% over 20 min). The fractions containing the product (retention time ~ 8.7 min) were collected, concentrated, and converted to an HCl salt according to general procedure (General remarks). Final compound **093** was obtained as a white solid (37 mg, 75%). **^{1}H NMR (400 MHz, D$_2$O)** δ 7.85 (s, 1H), 7.41 (d, *J* = 10.7 Hz, 1H), 7.01 (d, *J* = 7.2 Hz, 1H), 3.32 (t, *J* = 7.2 Hz, 2H), 2.93 (t, *J* = 7.2 Hz, 2H); **^{19}F NMR (376 MHz, D$_2$O)** δ -138.95 (dd, *J* = 10.8, 7.3 Hz); **^{13}C NMR (101 MHz, D$_2$O/CD$_3$OD)** δ 165.3, 151.3, 151.0, 149.0, 148.9,

148.6, 144.2, 144.1, 121.4, 114.8, 114.6, 113.0, 112.9, 105.7, 105.7, 39.0, 29.3; **HR-MS** calcd. for $C_{11}H_{11}FNO_3$ $[M+H]^+$ 224.0717, found 224.0722.

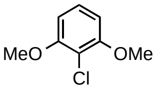

7a

2-Chloro-1,3-dimethoxybenzene (**7a**)

To a suspension of **6a** (0.85 g, 5.9 mmol) and freshly dry K_2CO_3 (1.8 g, 13 mmol) in dry acetone (35 ml) was added MeI (1.5 mL, 23.6 mmol) and the reaction flask was sealed with a teflon cap. The resulting mixture was heated to 50 °C for 20 h. Inorganic salts were removed by filtration, washed with acetone and the solution was concentrated. The crude product was dissolved in boiling hexane (~20 mL) and insoluble solid was filtered and washed with hot hexane (~2 x 5 mL). After cooling to 0 °C the compound **7a** crystalized as a white prism (0.92 g, 91%). **^{1}H NMR (400 MHz, Acetone-*d*$_6$)** δ 7.23 (t, *J* = 8.4 Hz, 1H), 6.74 (d, *J* = 8.4 Hz, 2H), 3.87 (s, 6H).[6]

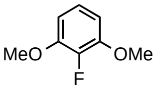

7b

2-Fluoro-1,3-dimethoxybenzene (**7b**)

To a suspension of **6b** (1.0 g, 7.80 mmol) and freshly dry K_2CO_3 (1.97 g, 14.1 mmol) in dry acetone (35 ml) was added MeI (1.6 mL, 25.6 mmol) and the reaction flask was sealed with a teflon cap. The resulting mixture was heated to 50 °C for 20 h. Inorganic salts were removed by filtration, washed with acetone and the solution was concentrated. The crude product was dissolved in boiling hexane (~20 mL) and insoluble solid was filtered, washed with hot hexane (~2 x 5 mL) and the solution was again concentrated. The crude product was purified by column chromatography (hexane:ethyl acetate – 7:1). The product **7b** was obtained as a colorless oil (1.1 g, 90%). **^{1}H NMR (500 MHz, Acetone-*d*$_6$)** δ 7.02 (td, *J* = 8.5, 2.3 Hz, 1H), 6.73 (dd, *J* = 8.4, 7.4 Hz, 2H), 3.85 (s, 6H).[7]

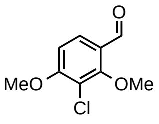

8a

3-Chloro-2,4-dimethoxybenzaldehyde (**8a**)

The compound **8a** was prepared from **7a** (750 mg, 4.35 mmol) in the same way as compound **2**. The crude product was purified by column chromatography (hexane:ethyl acetate – 7:1). The product **8a** was obtained as a white solid (95 mg, 12%; 560 mg of starting material was recovered). **^{1}H NMR (400 MHz,**

Acetone-*d*$_6$) δ 10.20 (d, *J* = 0.6 Hz, 1H), 7.77 (d, *J* = 8.8 Hz, 1H), 7.11 (d, *J* = 8.8 Hz, 1H), 4.03 (s, 3H), 4.01 (s, 3H).[8]

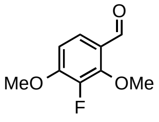

8b

3-Fluoro-2,4-dimethoxybenzaldehyde (**8b**)

The compound **8b** was prepared from **7b** (1.0 g, 6.4 mmol) in the same way as compound **2**. The crude product was purified by column chromatography (hexane:ethyl acetate – 7:1). The product **8b** was obtained as a white solid (570 mg, 48%; 105 mg of starting material was recovered). **^{1}H NMR (400 MHz, Acetone-*d*$_6$)** δ 10.20 (s, 1H), 7.57 (dd, *J* = 8.9, 2.1 Hz, 1H), 7.03 (app t, *J* = 7.9 Hz, 1H), 4.09 (d, *J* = 2.2 Hz, 3H), 4.00 (s, 3H).[9]

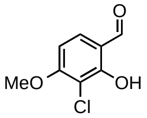

9a

3-Chloro-2-hydroxy-4-methoxybenzaldehyde (**9a**)

The compound **9a** was prepared from **8a** (90 mg, 0.45 mmol) in the same way as compound **3** in 3 h. The crude product was purified by column chromatography (CH_2Cl_2:hexane – 1:1). The product **9a** was obtained as a white solid (70 mg, 84%).**^{1}H NMR (400 MHz, Acetone-*d*$_6$)** δ 11.67 (br s, 1H), 9.87 (s, 1H), 7.77 (d, *J* = 8.8 Hz, 1H), 6.92 (d, *J* = 8.8 Hz, 1H), 4.04 (s, 3H).[10]

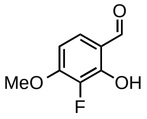

9b

3-Fluoro-2-hydroxy-4-methoxybenzaldehyde (**9b**)

The compound **9b** was prepared from **8b** (520 mg, 2.82 mmol) in the same way as compound **3** in 2 h. The crude product was purified by column chromatography (CH_2Cl_2:hexane – 1:1). The product **9a** was obtained as a white solid (70 mg, 84%). **^{1}H NMR (400 MHz, Acetone-*d*$_6$)** δ 11.10 (br s, 1H), 9.90 (d, *J* = 1.8 Hz, 1H), 7.59 (dd, *J* = 8.8, 2.0 Hz, 1H), 6.90 (dd, *J* = 8.8, 7.0 Hz, 1H), 4.01 (s, 3H); **^{19}F NMR (376 MHz, Acetone-*d*$_6$)** δ -161.89 (dt, *J* = 7.0, 1.9 Hz); **^{13}C NMR (101 MHz, Acetone-*d*$_6$)** δ 196.6, 196.5, 155.4, 155.3, 151.0, 150.9, 141.9, 139.5, 130.9, 130.9, 117.6, 117.6, 105.6, 57.1; **HR-MS** calcd. for $C_8H_6FO_3$ $[M-H]^-$ 169.0301, found 169.0310.

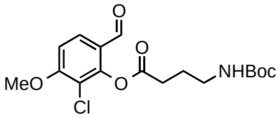

10a

2-Chloro-6-formyl-3-methoxyphenyl 4-((*tert*-butoxycarbonyl) amino)butanoate (**10a**)

The compound **10a** was prepared from **9a** (60 mg, 0.32 mmol) in

the same way as compound **4**. The crude product was purified by column chromatography (CH_2Cl_2 + 3% acetone). The product **10a** was obtained as a white solid (105 mg, 88%). **^{1}H NMR (400 MHz, Acetone-*d*$_6$)** δ 9.99 (s, 1H), 7.89 (d, *J* = 8.8 Hz, 1H), 7.27 (d, *J* = 8.8 Hz, 1H), 6.12 (br s, 1H), 4.07 (s, 3H), 3.23 (q, *J* = 6.6 Hz, 2H), 2.82 (q, *J* = 6.1, 5.2 Hz, 2H, overlaps with acetone residual peak), 1.99 – 1.91 (m, 2H), 1.41 (s, 9H); ^{13}C NMR (101 MHz, Acetone) δ 188.2, 171.2, 161.5, 156.8, 131.5, 124.2, 110.6, 78.6, 57.5, 40.2, 31.5, 30.1, 28.6, 26.0; **HR-MS** calcd. for $C_{17}H_{22}ClNNaO_6$ $[M+Na]^+$ 394.1033, found 394.1044.

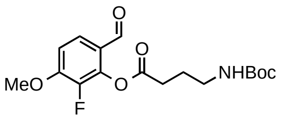

10b

2-Fluoro-6-formyl-3-methoxyphenyl 4-((*tert*-butoxycarbonyl) amino)butanoate (**10b**)

The compound **10b** was prepared from **9b** (230 mg, 1.35 mmol) in the same way as compound **4**. The crude product was purified by column chromatography (CH_2Cl_2 + 3% acetone). The product **10b** was obtained as a white solid (445 mg, 93%). **^{1}H NMR (400 MHz, Acetone-*d*$_6$)** δ 10.01 (s, 1H), 7.73 (dd, *J* = 8.8, 2.0 Hz, 1H), 7.27 (dd, *J* = 8.6, 7.6 Hz, 1H), 6.11 (br s, 1H), 4.04 (s, 3H), 3.22 (q, *J* = 6.6 Hz, 2H), 2.81 (t, *J* = 7.4 Hz, 2H overlaps with acetone residual peak), 1.94 (p, *J* = 7.1 Hz, 3H), 1.41 (s, 9H); **^{19}F NMR (376 MHz, Acetone-*d*$_6$)** δ -151.00 (d, *J* = 7.1 Hz); **^{13}C NMR (101 MHz, Acetone-*d*$_6$)** δ 188.3, 188.3, 171.2, 156.8, 154.5, 154.4, 146.4, 144.0, 127.8, 123.7, 111.4, 100.9, 78.6, 57.3, 40.2, 31.4, 28.6, 26.0; **HR-MS** calcd. for $C_{17}H_{22}FNNaO_6$ $[M+Na]^+$ 378.1329, found 378.1331.

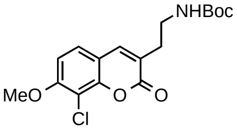

11a

***tert*-Butyl (2-(8-chloro-7-methoxy-2-oxo-2*H*-chromen-3-yl)ethyl)carbamate** (**11a**)

The compound **11a** was prepared from **10a** (100 mg, 0.27 mmol) in the same way as compound **5**. The crude product was purified by column chromatography (CH_2Cl_2 + 2% acetone). The product **11a** was obtained in mixture with Boc-GABA-OH (~1:3) as a colorless viscous oil and was used in the final step without further purification (25 mg, 26%, of **11a** based on an ^{1}H NMR ratio). **^{1}H NMR (400 MHz, Acetone-*d*$_6$)** δ 7.69 (s, 1H), 7.57 (d, *J* = 8.7 Hz, 1H), 7.15 (d, *J* = 8.7 Hz, 1H), 6.12 (br s, 1H), 4.01 (s, 3H), 3.71 (t, J = 7.1 Hz, 6H)-GABA, 3.37 (q, J = 6.3 Hz, 2H), 2.68 (t,

J = 6.3 Hz, 2H), 2.40 (t, J = 8.1 Hz, 6H)-GABA, 1.98 (p, J = 7.6 Hz, 6H)-GABA, 1.47 (s, 27H)-GABA, 1.31 (s, 9H); **LR-MS** calcd. for $C_{12}H_{13}ClNO_3$ $[M+H-Boc]^+$ 254.06, found 254.11.

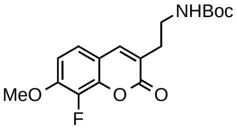

11b

tert-Butyl (2-(8-fluoro-7-methoxy-2-oxo-2*H*-chromen-3-yl)ethyl)carbamate (11b)

The compound **11b** was prepared from **10b** (430 mg, 1.21 mmol) in the same way as compound **5**. The crude product was purified by column chromatography (hexane:ethyl acetate – 5:2). The product **11b** was obtained as a white solid (165 mg, 40%). **^{1}H NMR (400 MHz, Acetone-*d*$_6$)** δ 7.69 (d, *J* = 0.9 Hz, 1H), 7.39 (dd, *J* = 8.8, 2.1 Hz, 1H), 7.14 (dd, *J* = 8.7, 7.5 Hz, 1H), 6.11 (br s, 1H), 3.99 (s, 3H), 3.37 (q, *J* = 6.3 Hz, 2H), 2.67 (td, *J* = 6.4, 0.8 Hz, 2H), 1.31 (s, 9H); **^{19}F NMR (376 MHz, Acetone-*d*$_6$)** δ -158.63 – -158.69 (m); **^{13}C NMR (101 MHz, Acetone-*d*$_6$)** δ 160.8, 156.7, 150.6, 150.5, 143.3, 143.2, 141.0, 140.5, 138.6, 125.2, 123.4, 123.3, 115.4, 110.0, 78.6, 57.2, 39.2, 32.8, 28.6; **HR-MS** calcd. for $C_{17}H_{20}FNNaO_5$ $[M+Na]^+$ 360.1223, found 360.1227.

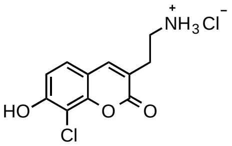

269

3-(2-Aminoethyl)-8-chloro-7-hydroxy-2*H*-chromen-2-one hydrochloride salt (269)

A solution of compound **11a** (15 mg, 0.042 mmol) in AcOH (0.5 mL) and HBr (0.5 mL) was heated in closed vial to 110 °C for 48 h. The reaction mixture was then concentrated and the crude product was purified by reverse phase HPLC using a linear gradient - water (A), MeOH (B) (25% B → 95% over 20 min). The fractions containing the product (retention time ~ 9.7 min) were collected, concentrated, and converted to an HCl salt according to general procedure (General remarks). Final compound **269** was obtained as a white solid (7 mg, 61%). **^{1}H NMR (400 MHz, D_2O)** δ 7.88 (s, 1H), 7.44 (d, *J* = 8.7 Hz, 1H), 7.01 (d, *J* = 8.6 Hz, 1H), 3.34 (t, *J* = 7.2 Hz, 2H), 2.94 (t, *J* = 7.1 Hz, 2H); **^{13}C NMR (101 MHz, D_2O/CD_3OD)** δ 164.2, 156.6, 150.9, 144.5, 128.1, 120.5, 114.4, 114.1, 107.8, 38.9, 29.2; **HR-MS** calcd. for $C_{11}H_{11}ClNO_3$ $[M+H]^+$ 240.0422, found 240.0428.

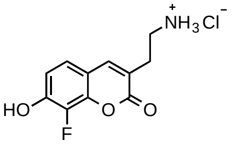

FFN270

3-(2-Aminoethyl)-8-fluoro-7-hydroxy-2*H*-chromen-2-one hydrochloride salt (FFN270)

A solution of compound **11b** (20 mg, 0.059 mmol) in AcOH (0.5 mL) and HBr (0.5 mL) was heated in closed vial to 110 °C for 48 h. The reaction mixture was then concentrated and the crude product was purified by reverse phase HPLC using a linear gradient - water (A), MeOH (B) (25% B → 95% over 20 min). The fractions containing the product (retention time ~ 8.4 min) were collected, concentrated, and converted to an HCl salt according to general procedure (General remarks). Final compound **FFN270** was obtained as a white solid (12 mg, 78%). **^{1}H NMR (400 MHz, D_2O)** δ 7.90 (s, 1H), 7.31 (dd, *J* = 8.7, 1.8 Hz, 1H), 7.01 (dd, *J* = 8.2, 0.5 Hz, 1H), 3.33 (t, *J* = 7.2 Hz, 2H), 2.94 (t, *J* = 7.2 Hz, 2H); **^{19}F NMR (376 MHz, D_2O)** δ -159.24 (dt, *J* = 7.7, 1.5 Hz); **^{13}C NMR (101 MHz, D_2O/CD_3OD)** δ 164.0, 148.1, 148.0, 144.5, 144.5, 143.2, 143.2, 140.3, 137.9, 124.3, 124.3, 120.9, 115.4, 114.3, 39.0, 29.4; **HR-MS** calcd. for $C_{11}H_{11}FNO_3$ $[M+H]^+$ 224.0717, found 224.0732.

Supplementary Figures 15-40. NMR Spectra.

Supplementary Figure 15. ^{1}H-NMR of compound **4**

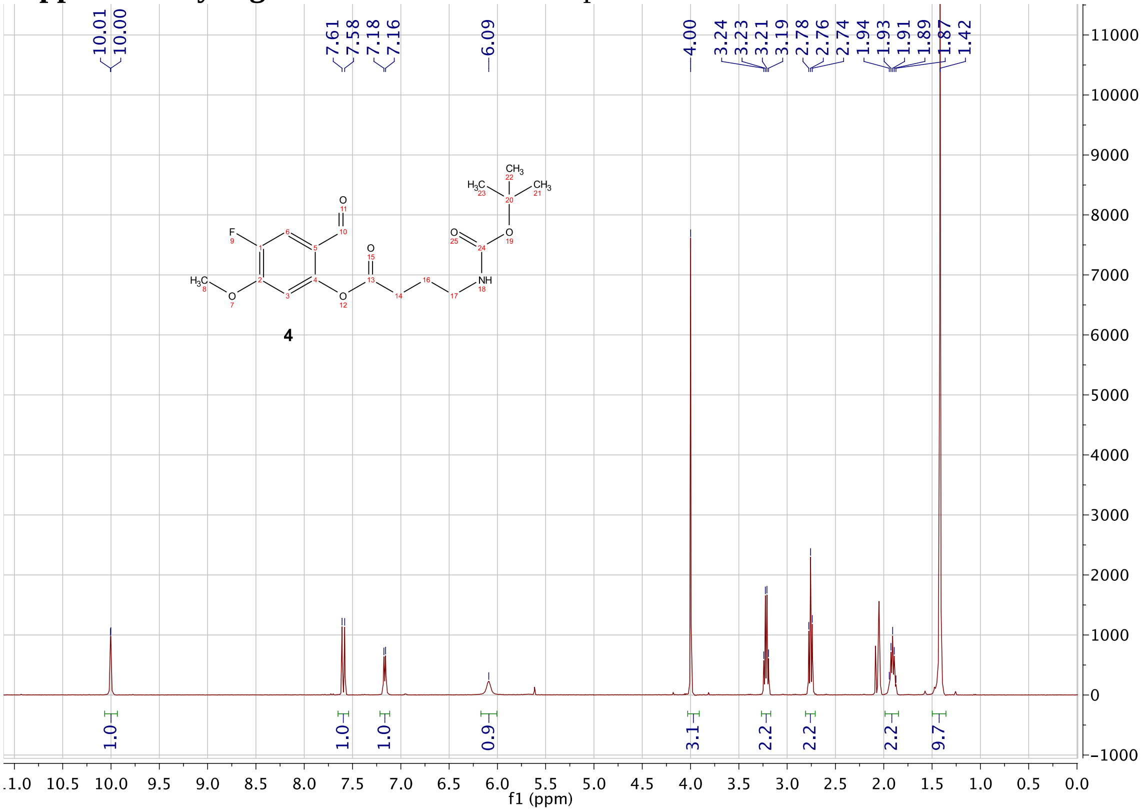

Supplementary Figure 16. ^{13}C-NMR of compound **4**

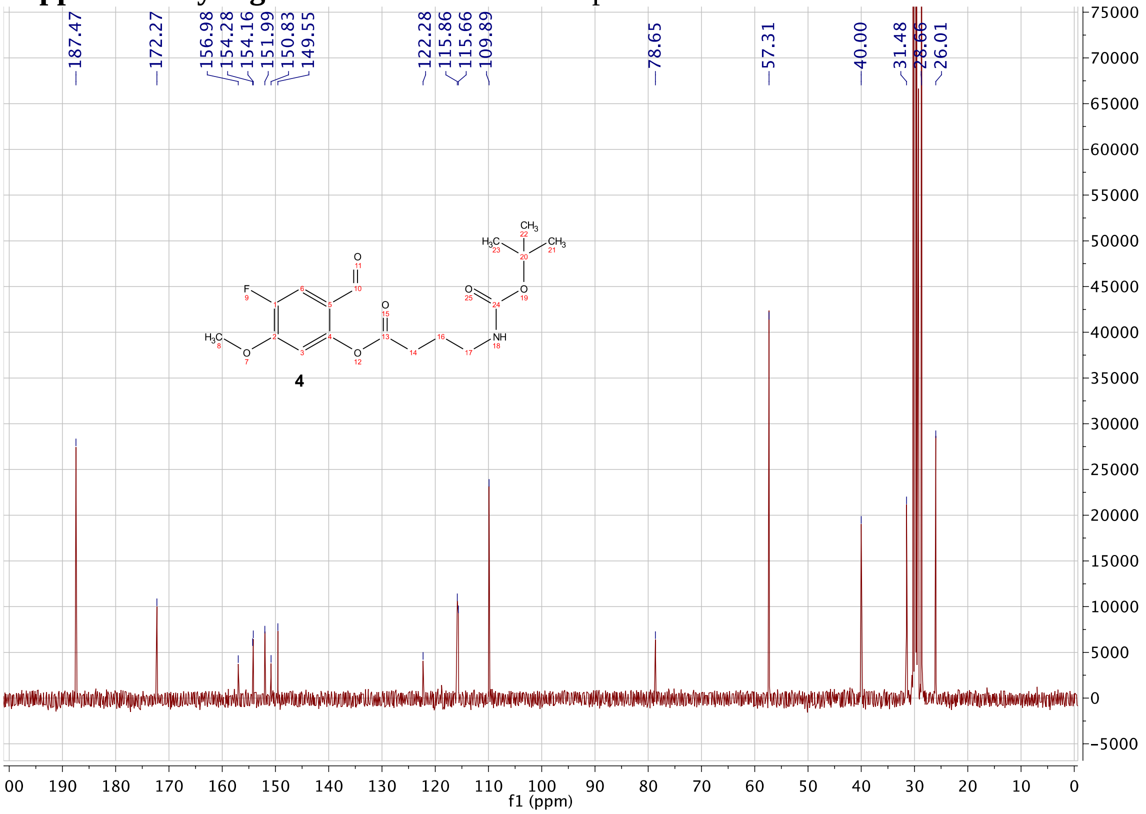

Supplementary Figure 17. ^{19}F-NMR of compound **4**

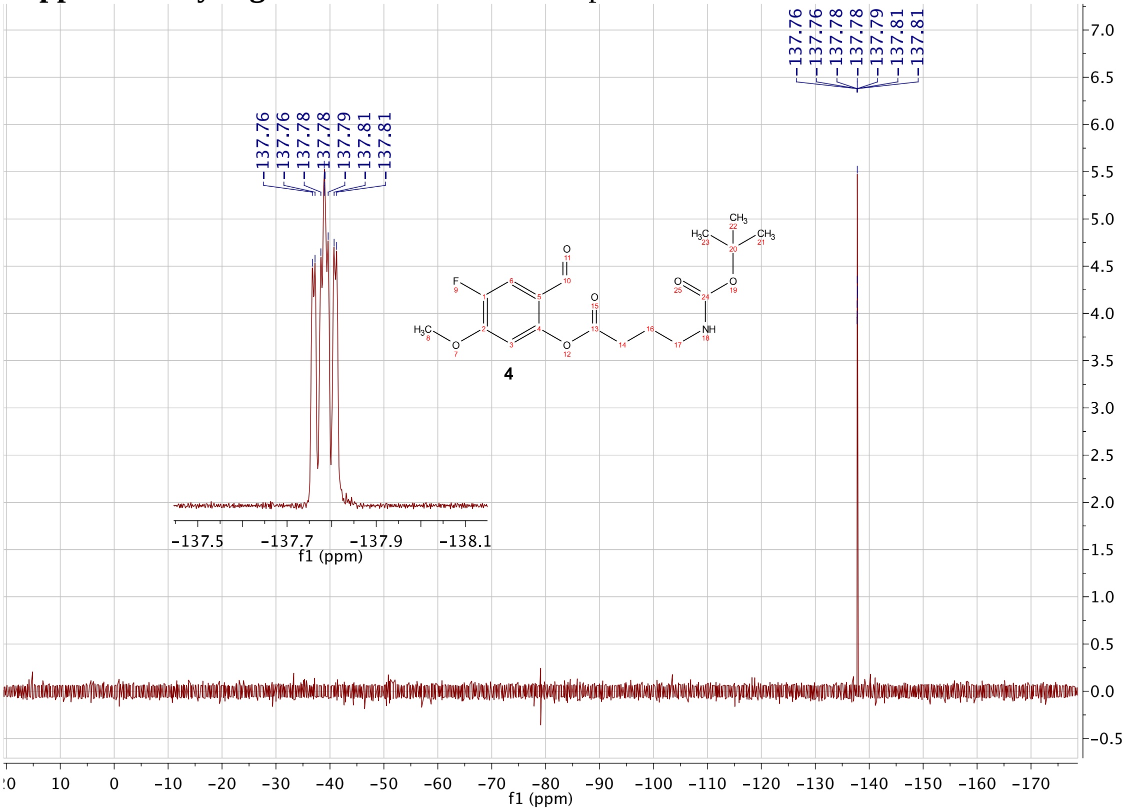

Supplementary Figure 18. ^{1}H-NMR of compound **5**

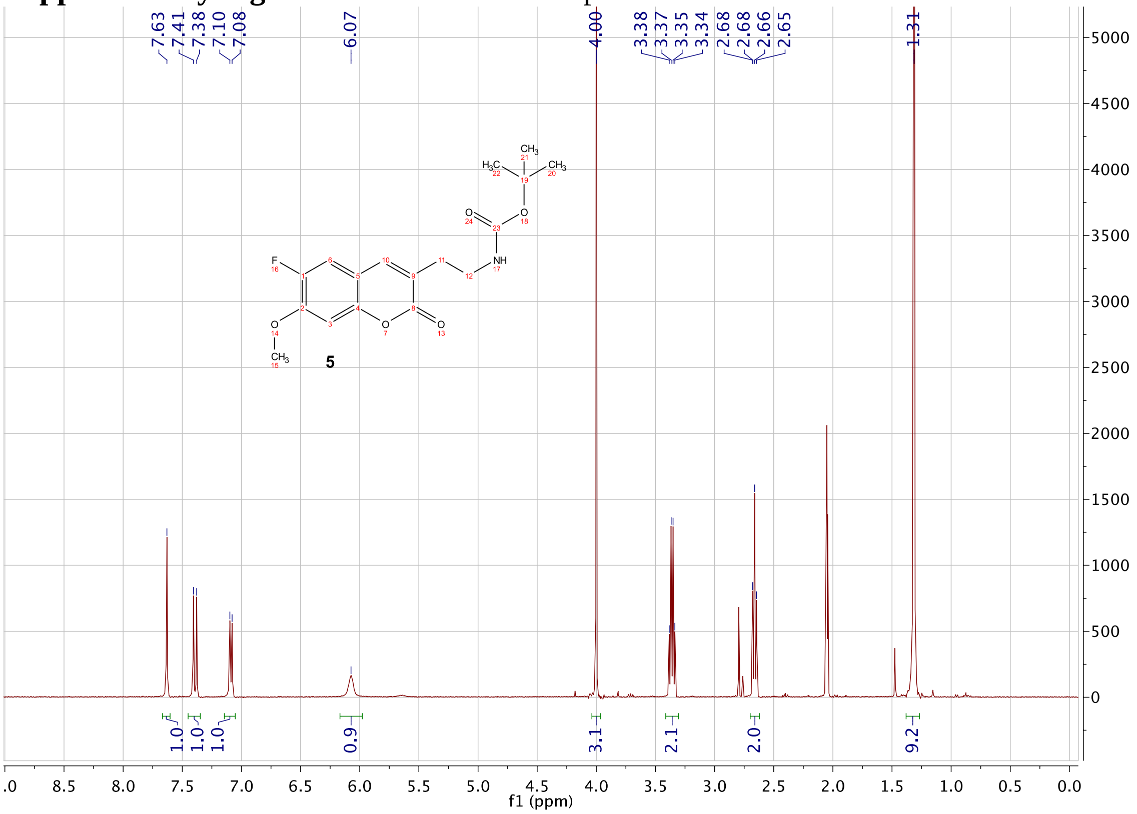

Supplementary Figure 19. ^{13}C-NMR of compound **5**

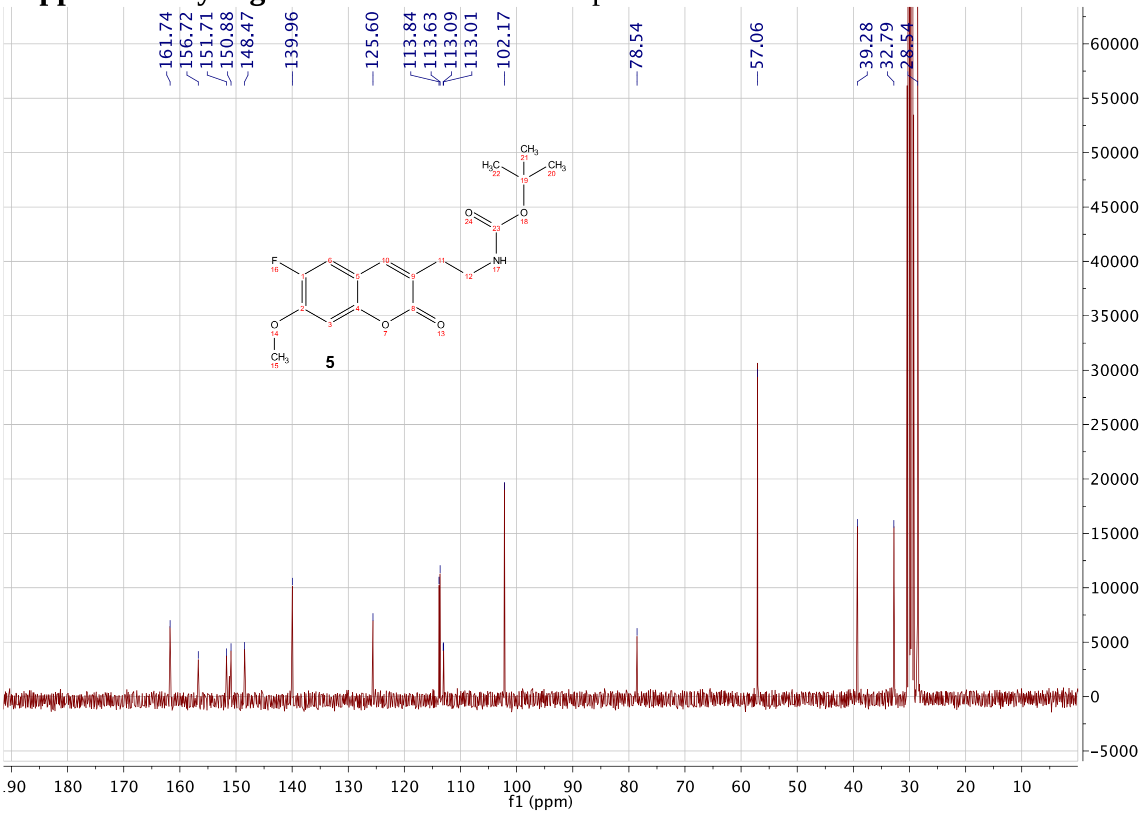

Supplementary Figure 20. ^{19}F-NMR of compound **5**

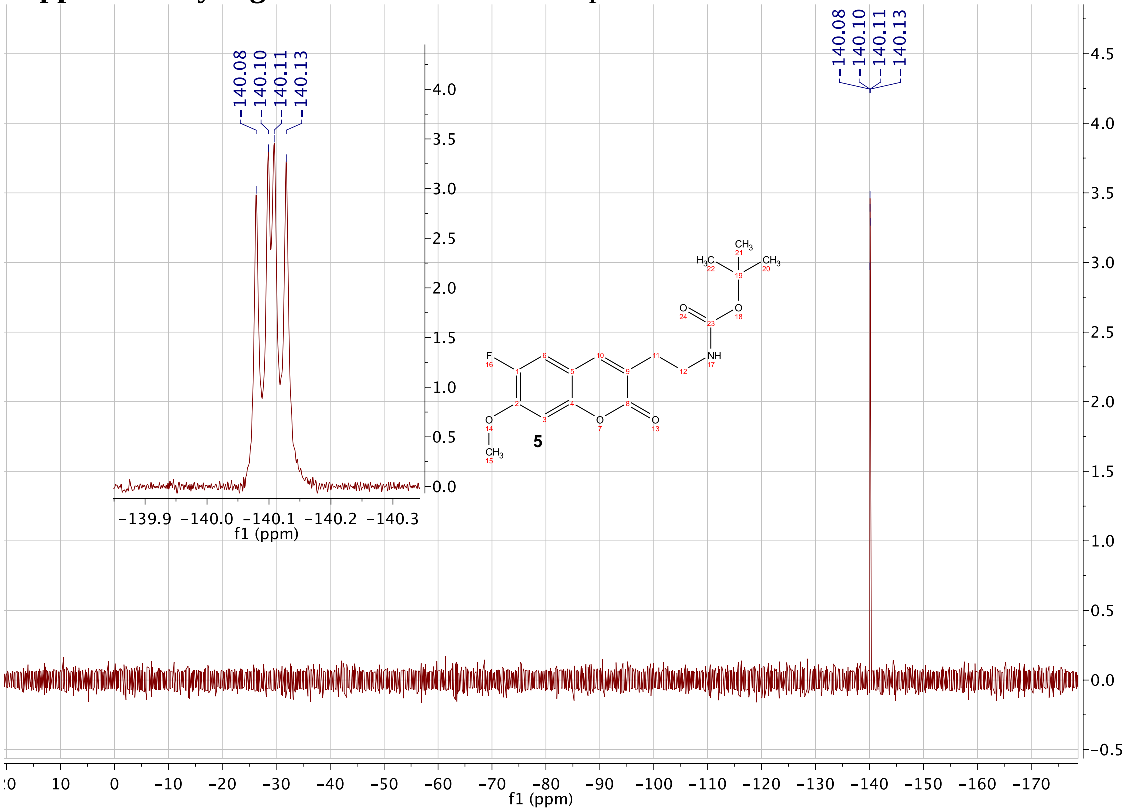

Supplementary Figure 21. ^{1}H-NMR of compound **093**

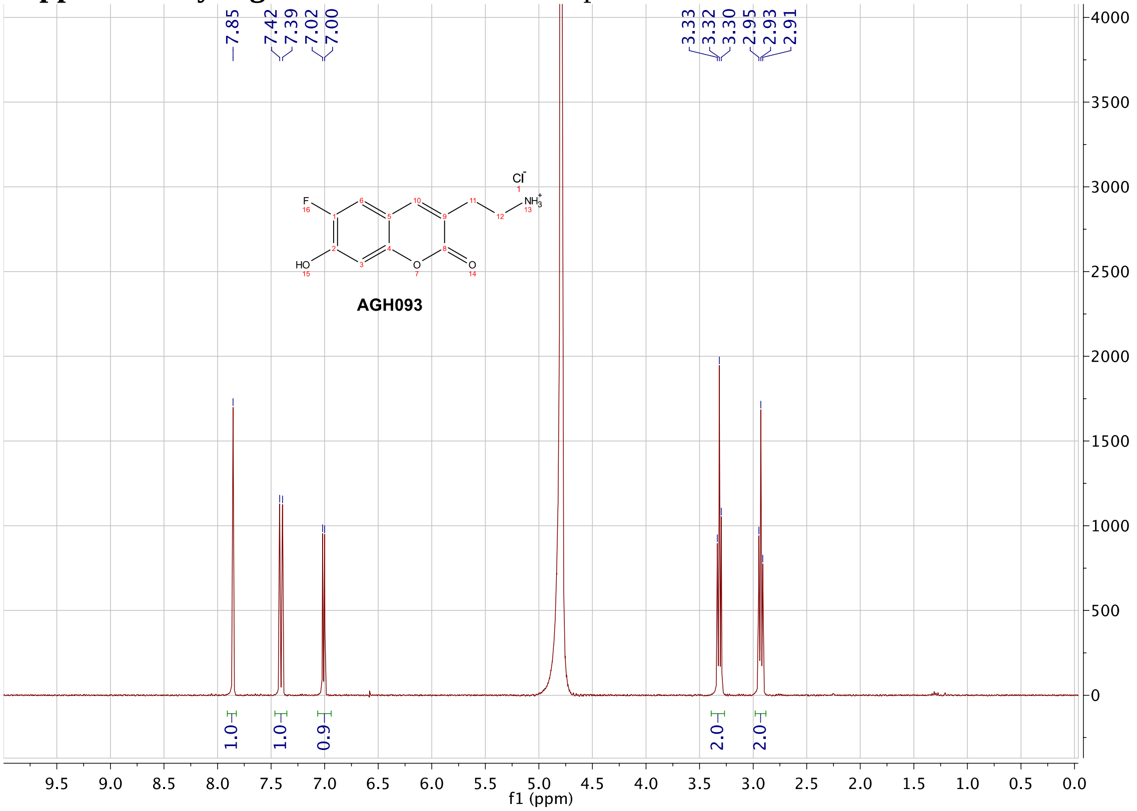

Supplementary Figure 22. ^{13}C-NMR of compound **093**

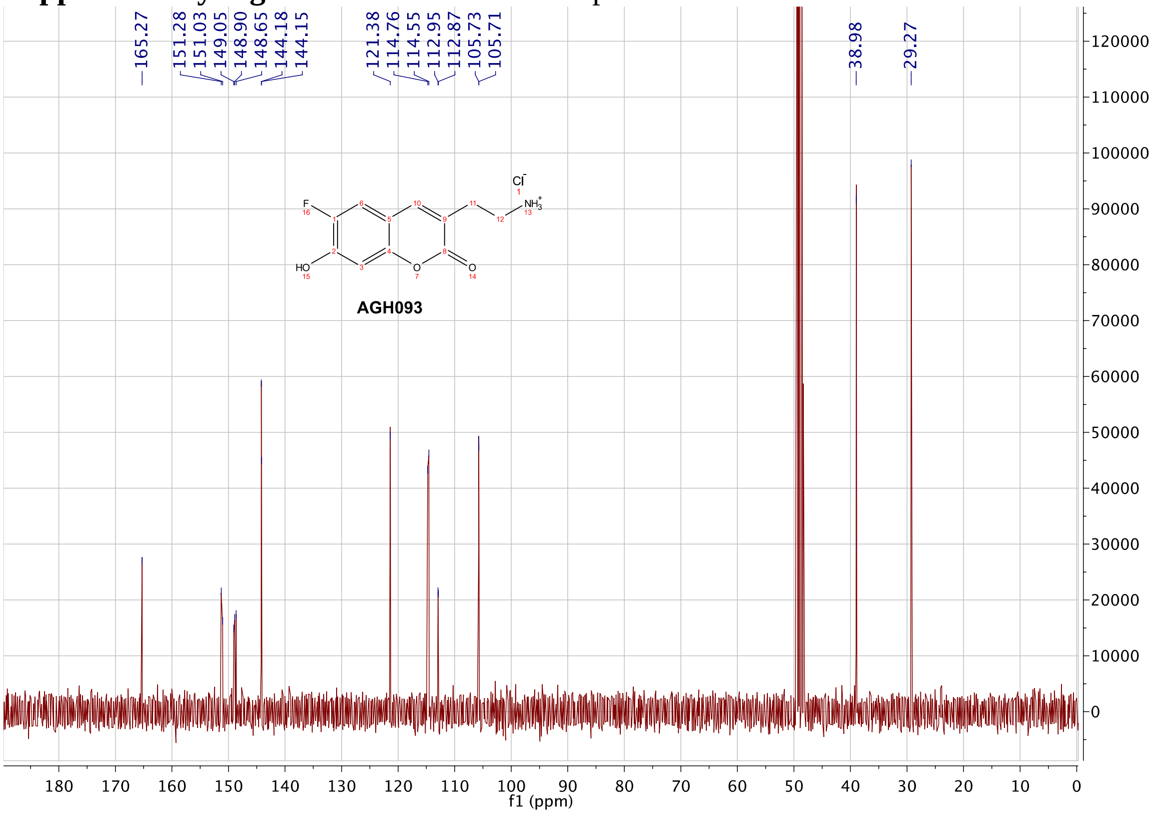

Supplementary Figure 23. ^{19}F-NMR of compound **093**

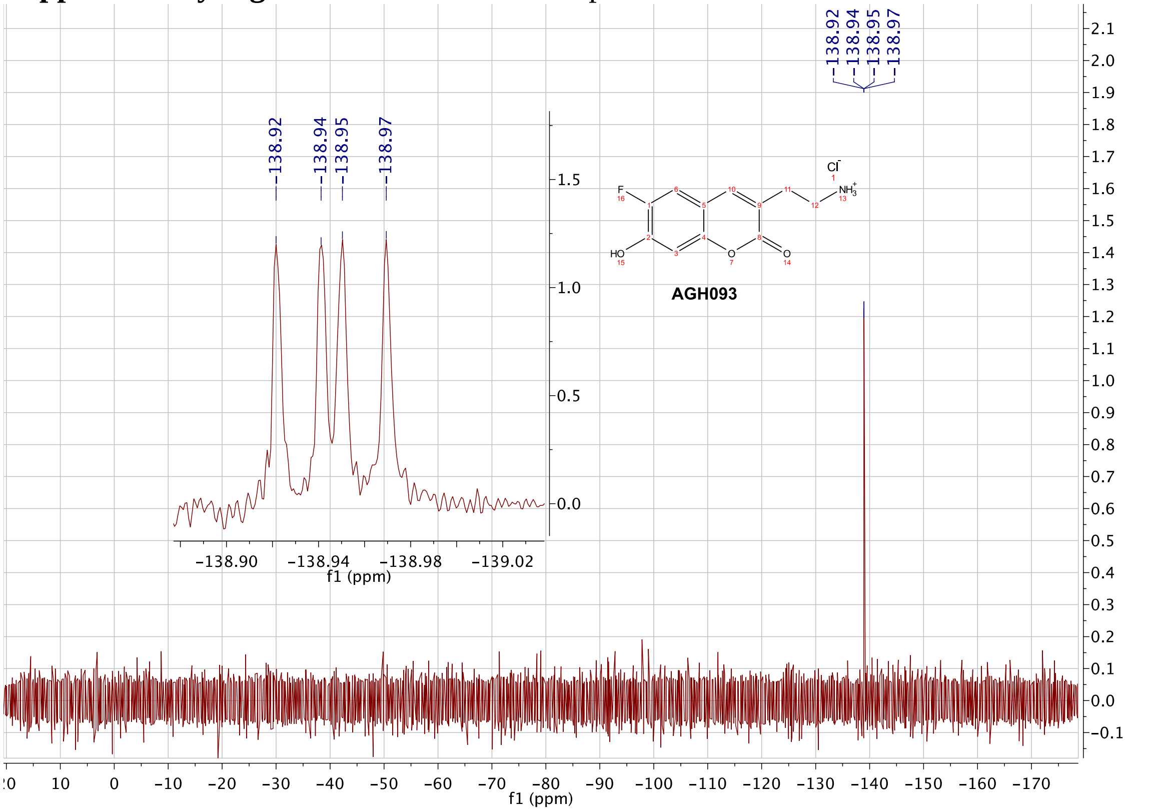

Supplementary Figure 24. ^{1}H-NMR of compound **9b**

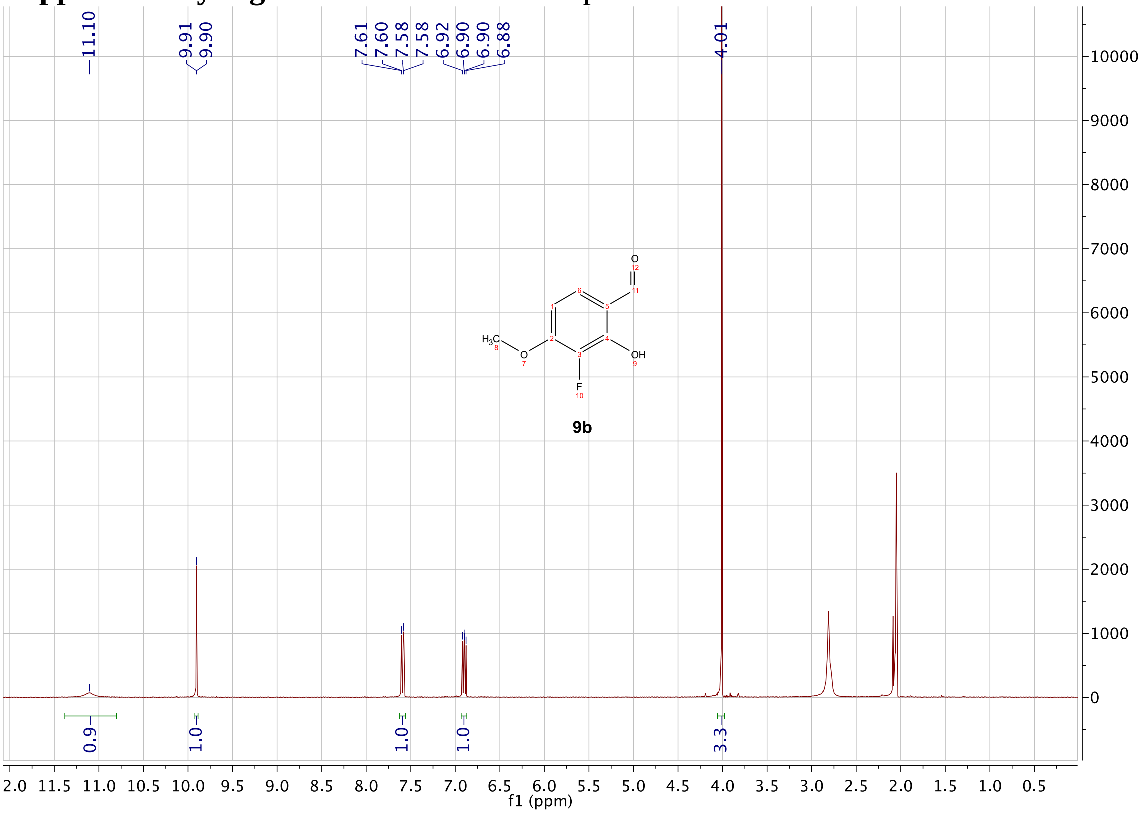

Supplementary Figure 25. ^{13}C-NMR of compound **9b**

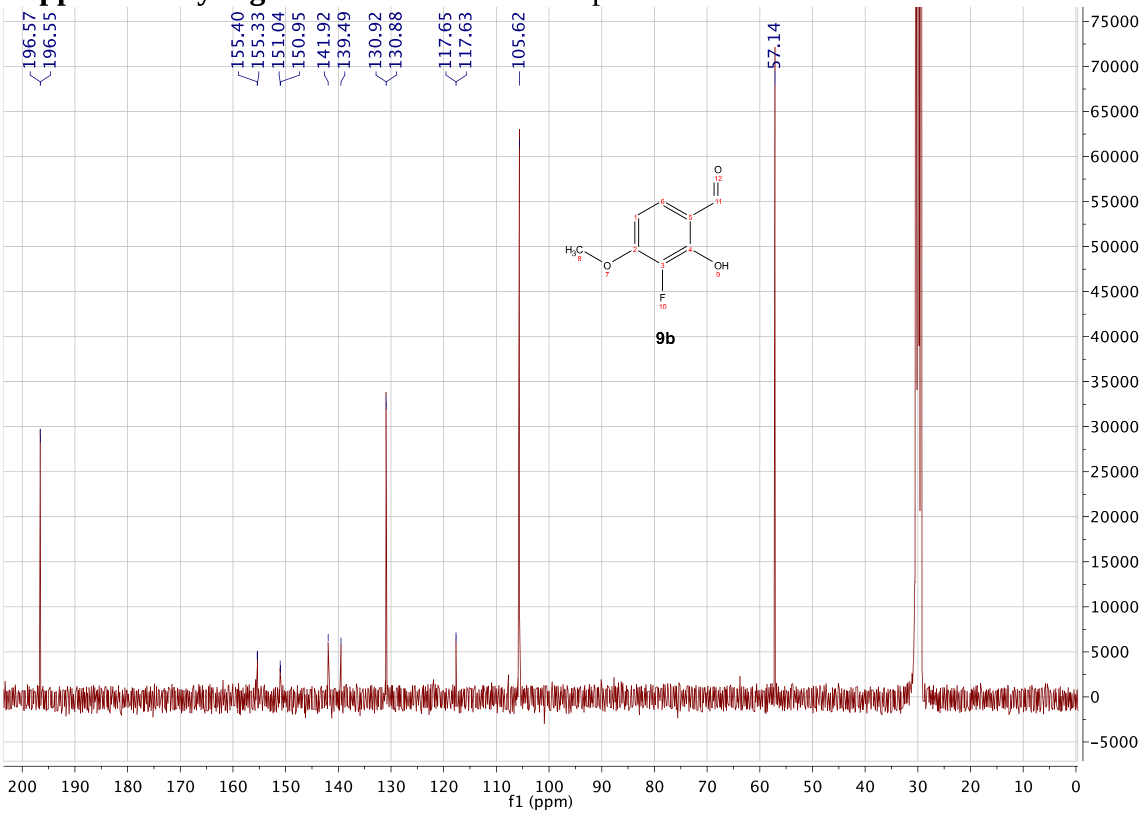

Supplementary Figure 26. ^{19}F-NMR of compound **9b**

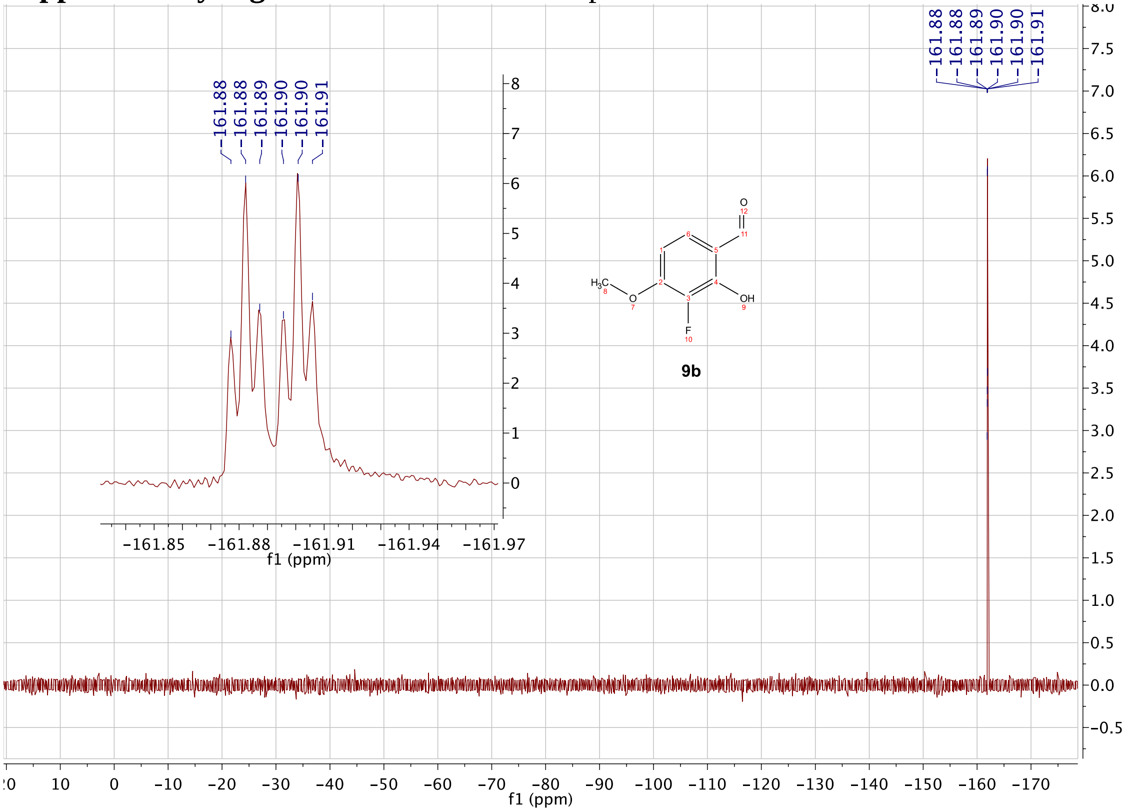

Supplementary Figure 27. ^{1}H-NMR of compound **10a**

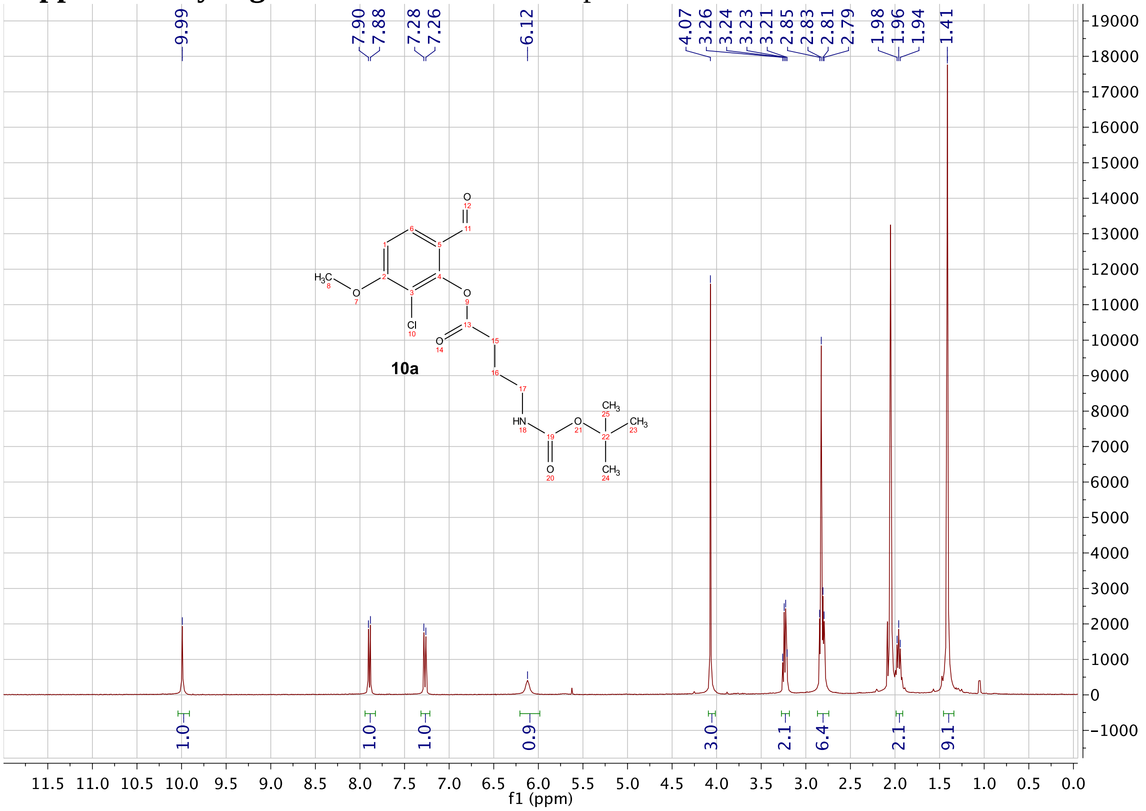

Supplementary Figure 28. ^{13}C-NMR of compound **10a**

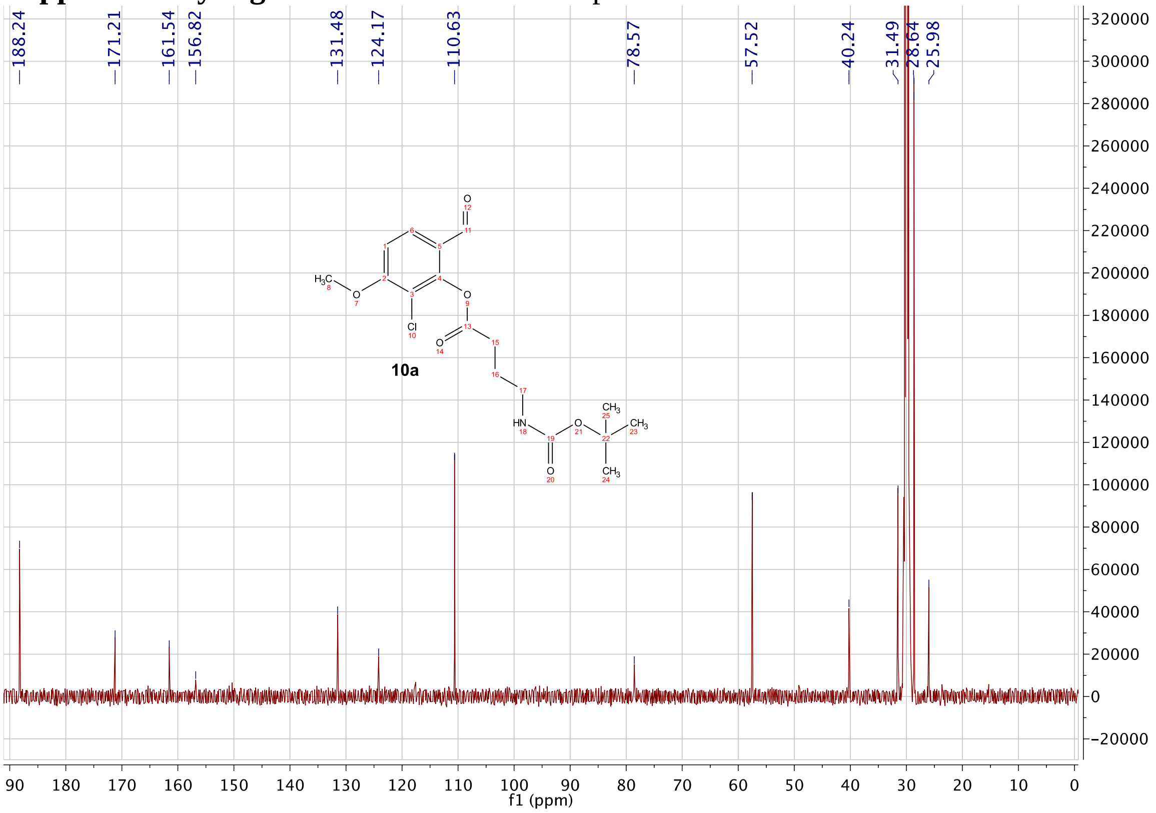

Supplementary Figure 29. ^{1}H-NMR of compound **10b**

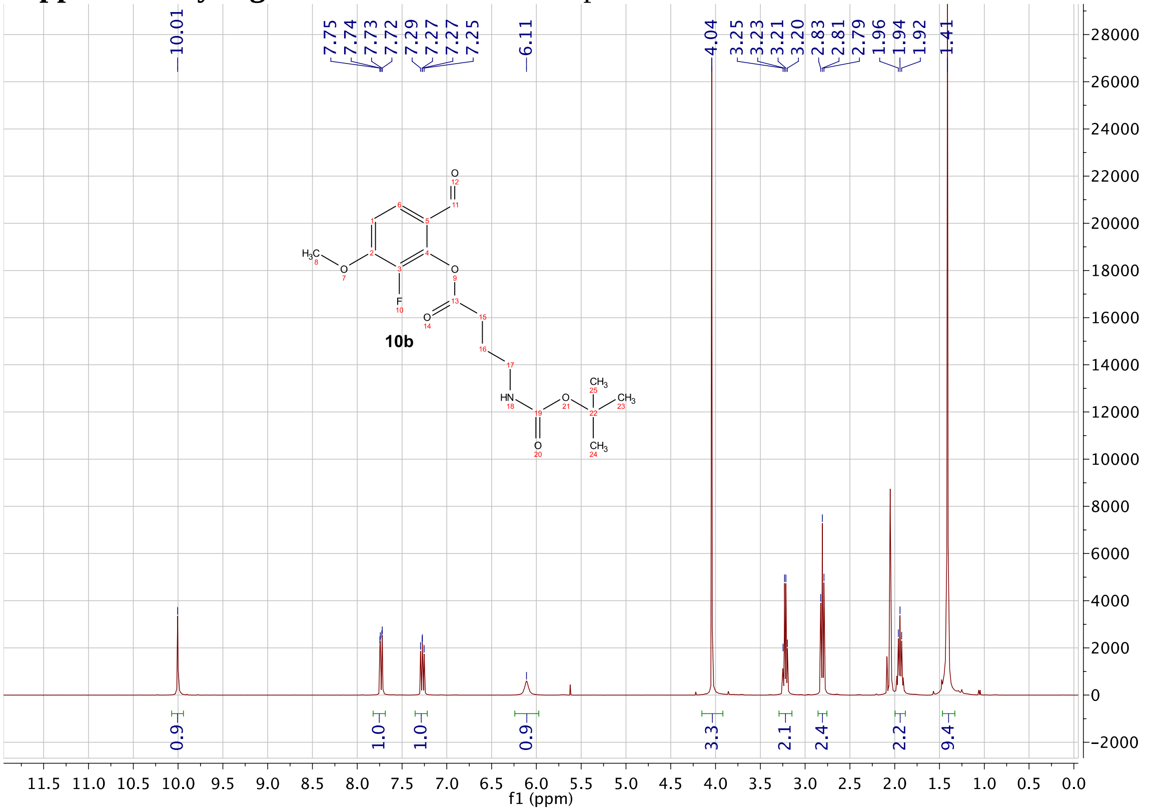

Supplementary Figure 30. ^{13}C-NMR of compound **10b**

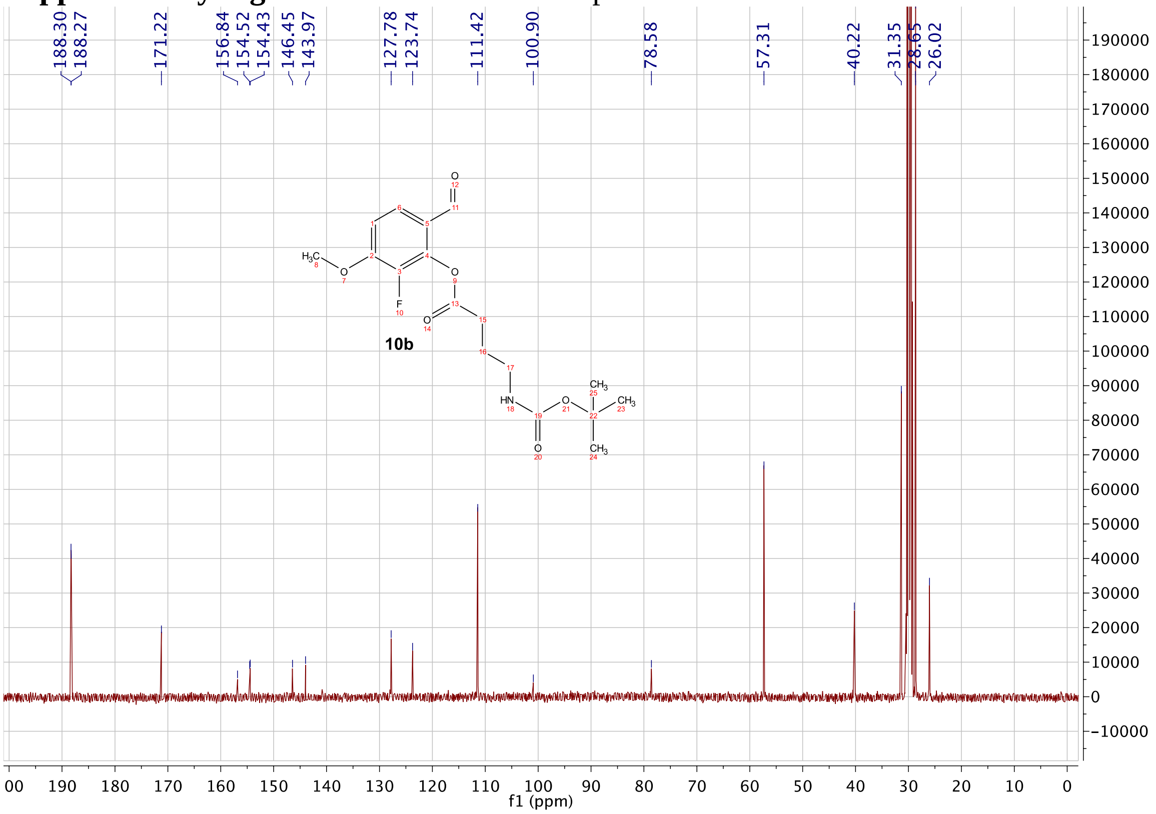

Supplementary Figure 31. ^{19}F-NMR of compound **10b**

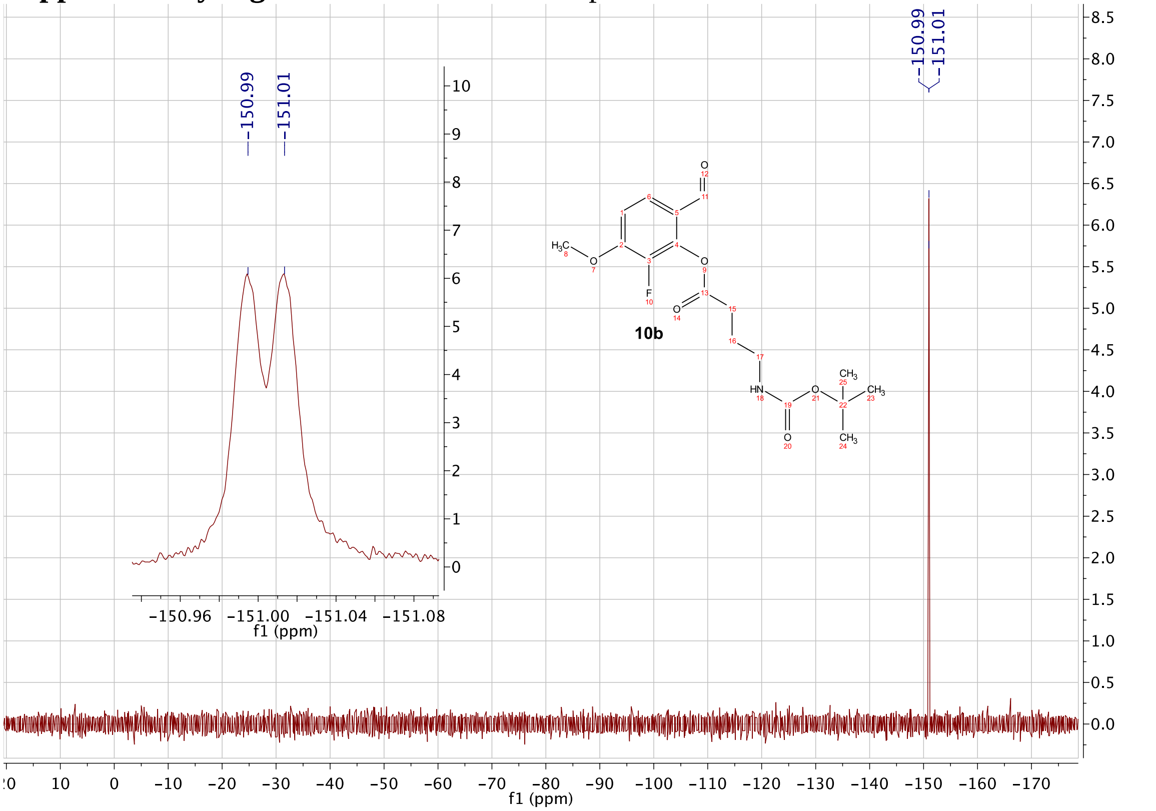

Supplementary Figure 32. ^{1}H-NMR of compound **11a**

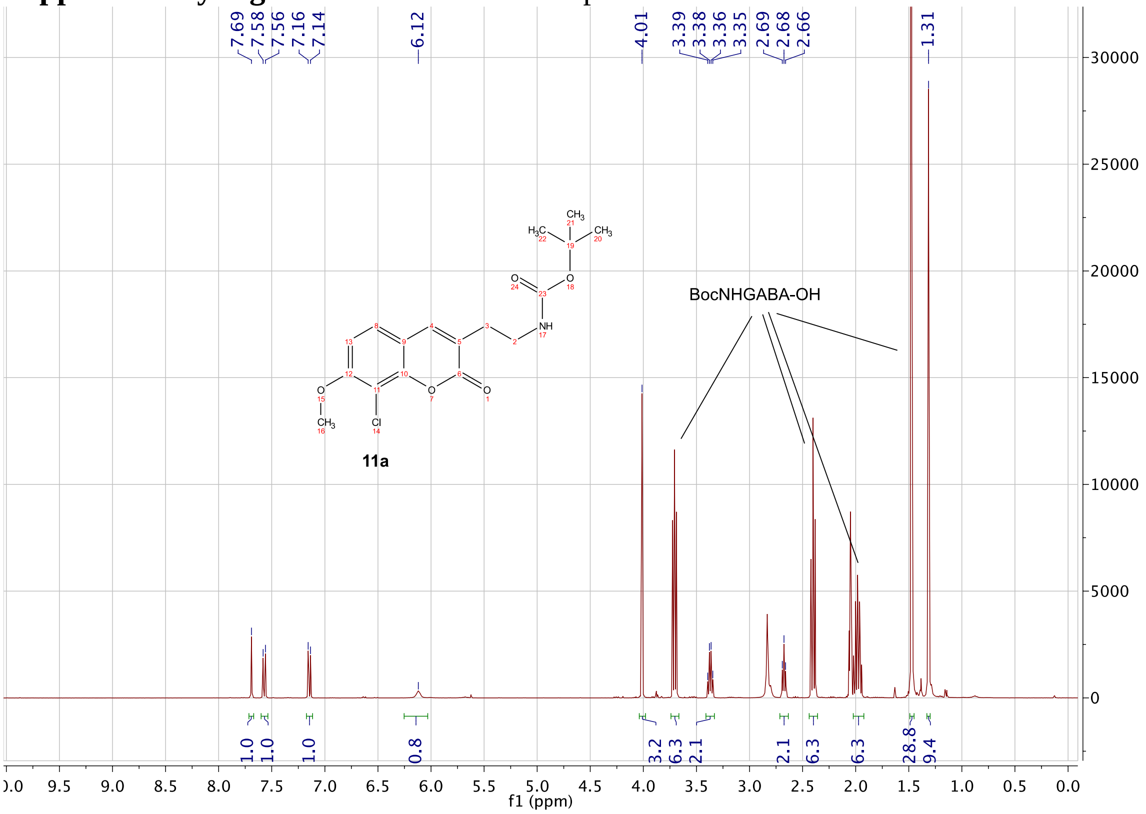

Supplementary Figure 33. ^{1}H-NMR of compound **11b**

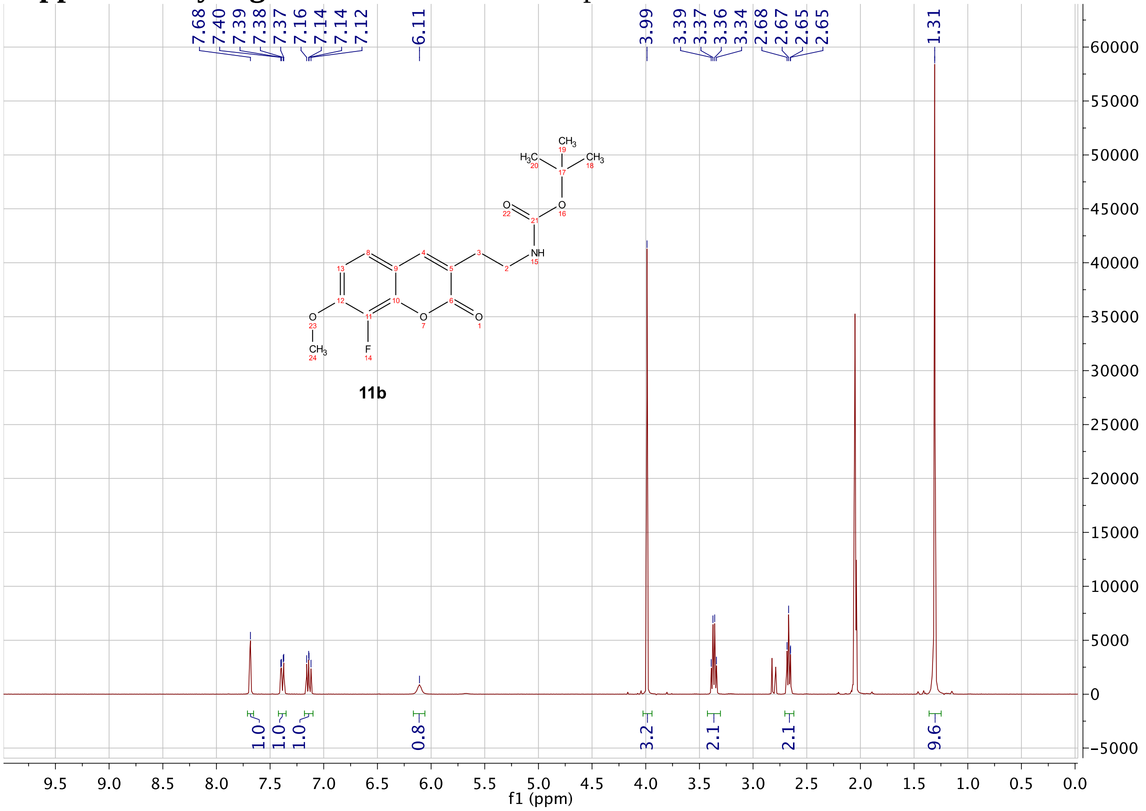

Supplementary Figure 34. ^{13}C-NMR of compound **11b**

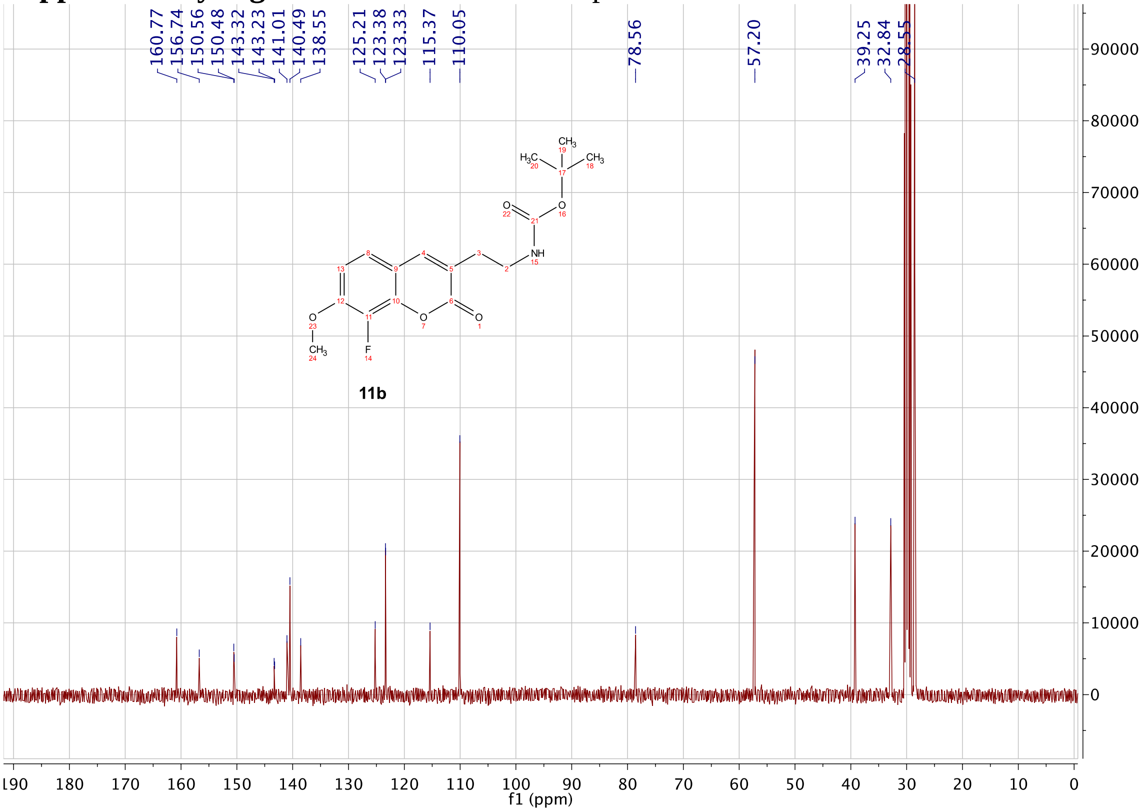

Supplementary Figure 35. ^{19}F-NMR of compound **11b**

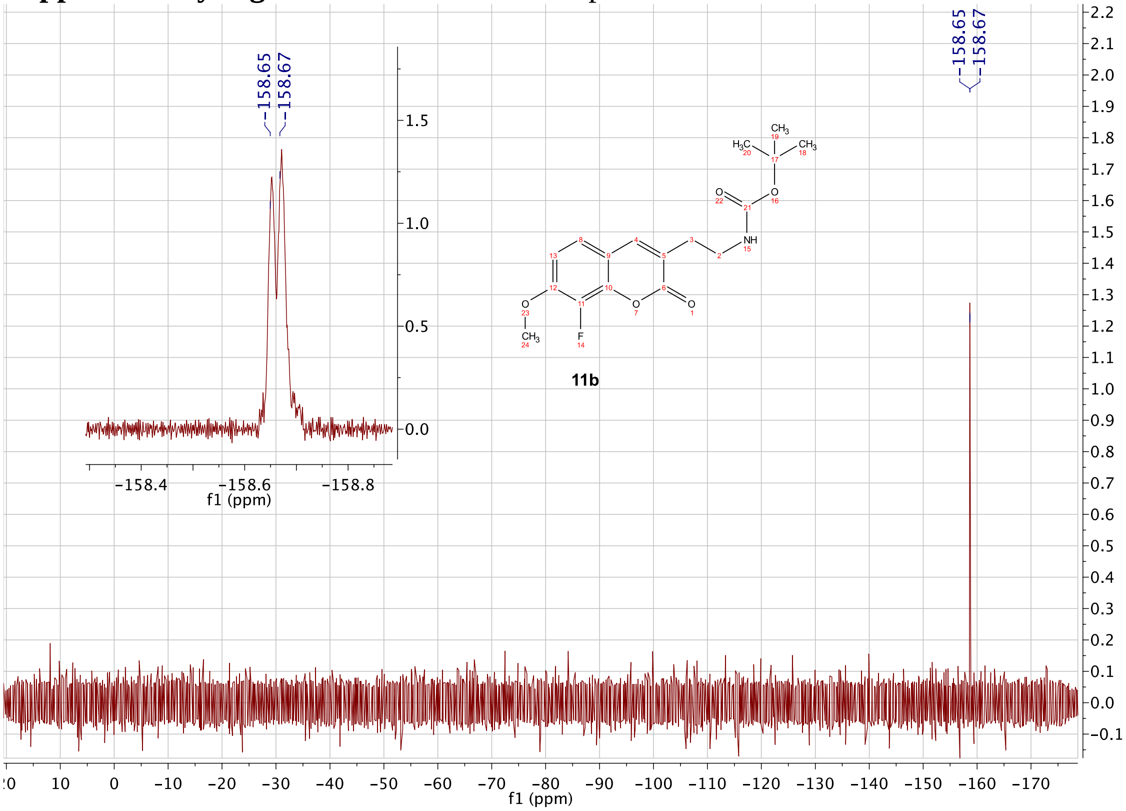

Supplementary Figure 36. ^{1}H-NMR of compound **269**

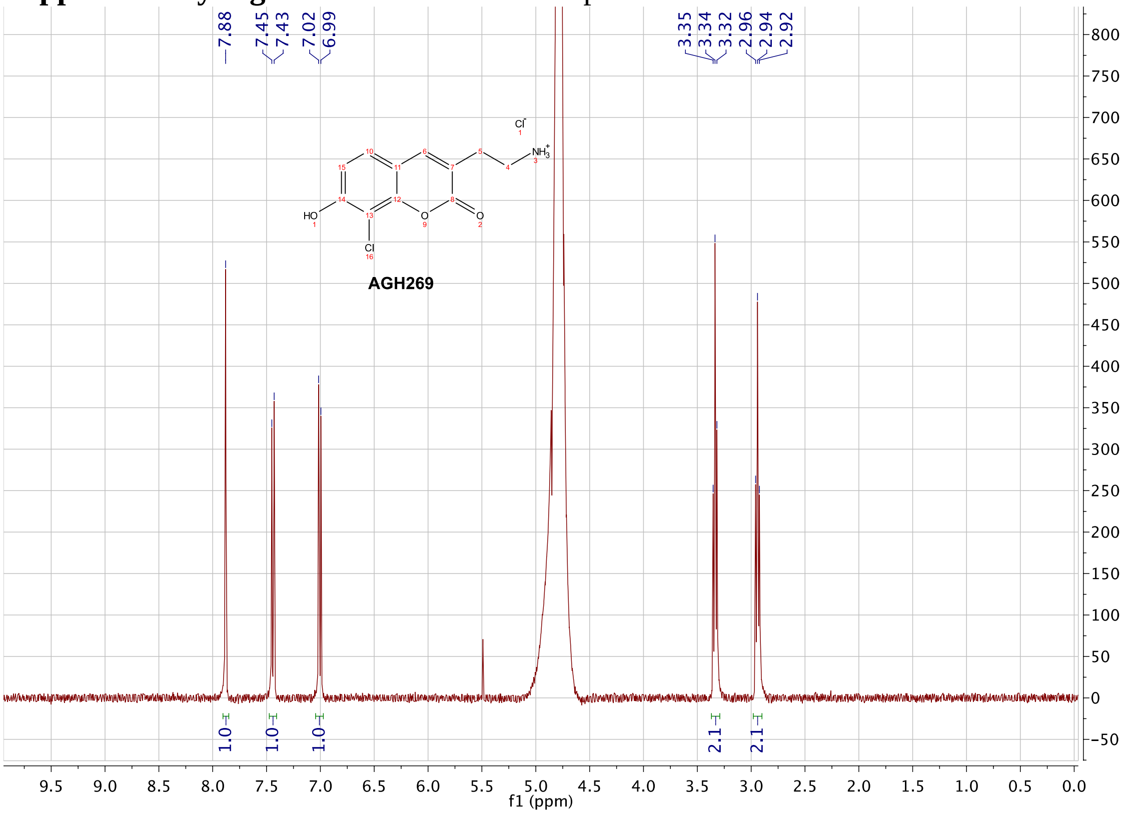

Supplementary Figure 37. ^{13}C-NMR of compound **269**

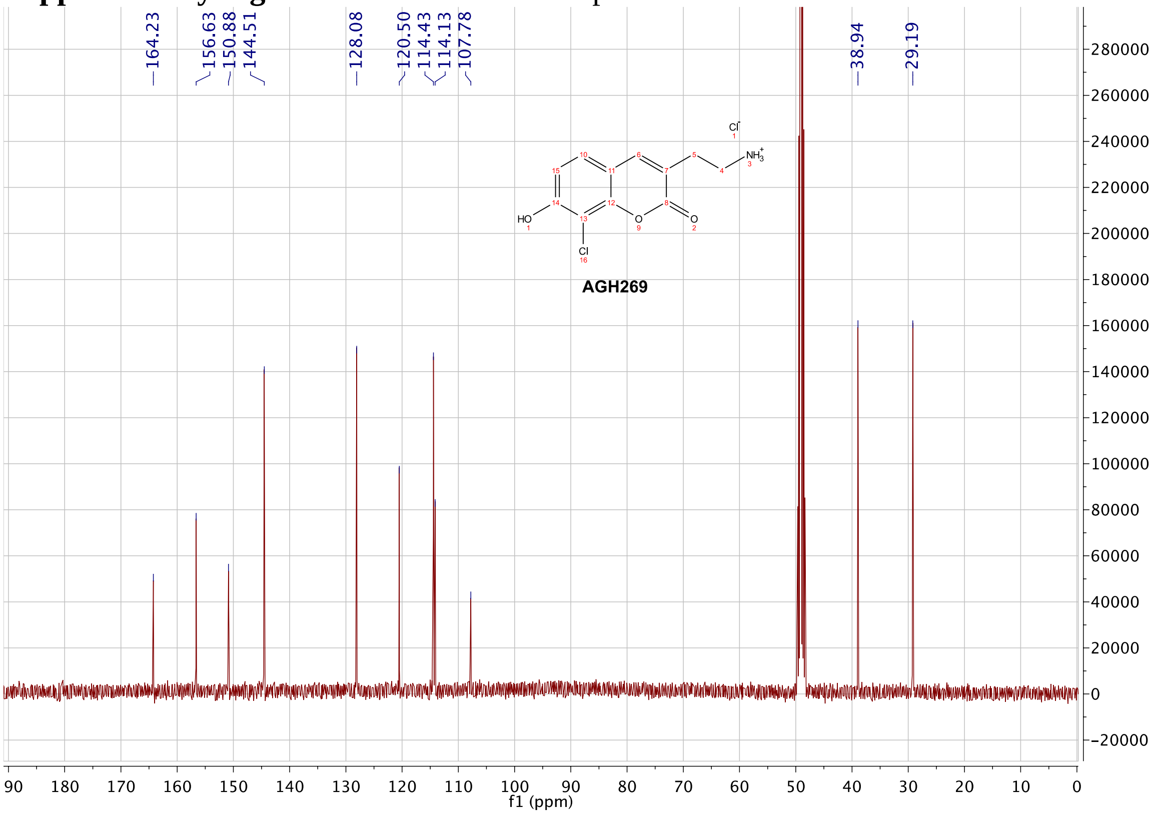

Supplementary Figure 38. ^{1}H-NMR of compound **FFN270**

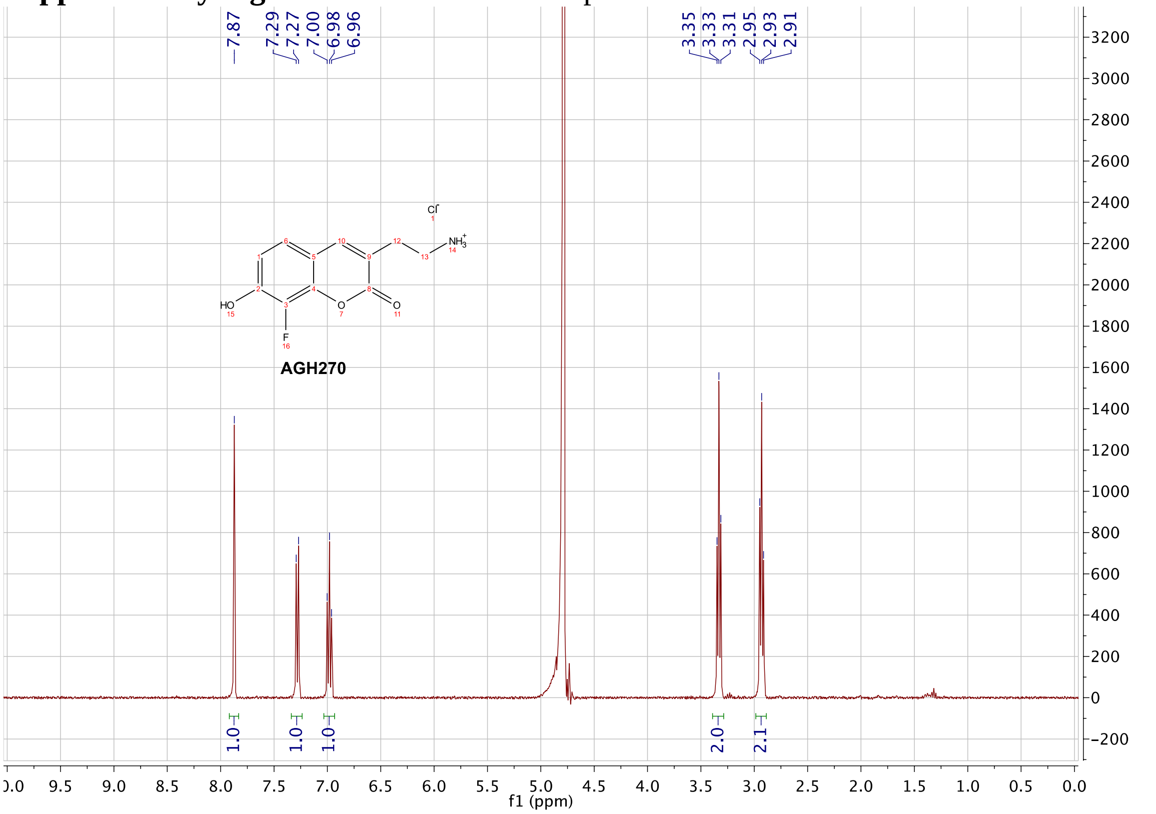

Supplementary Figure 39. ^{13}C-NMR of compound **FFN270**

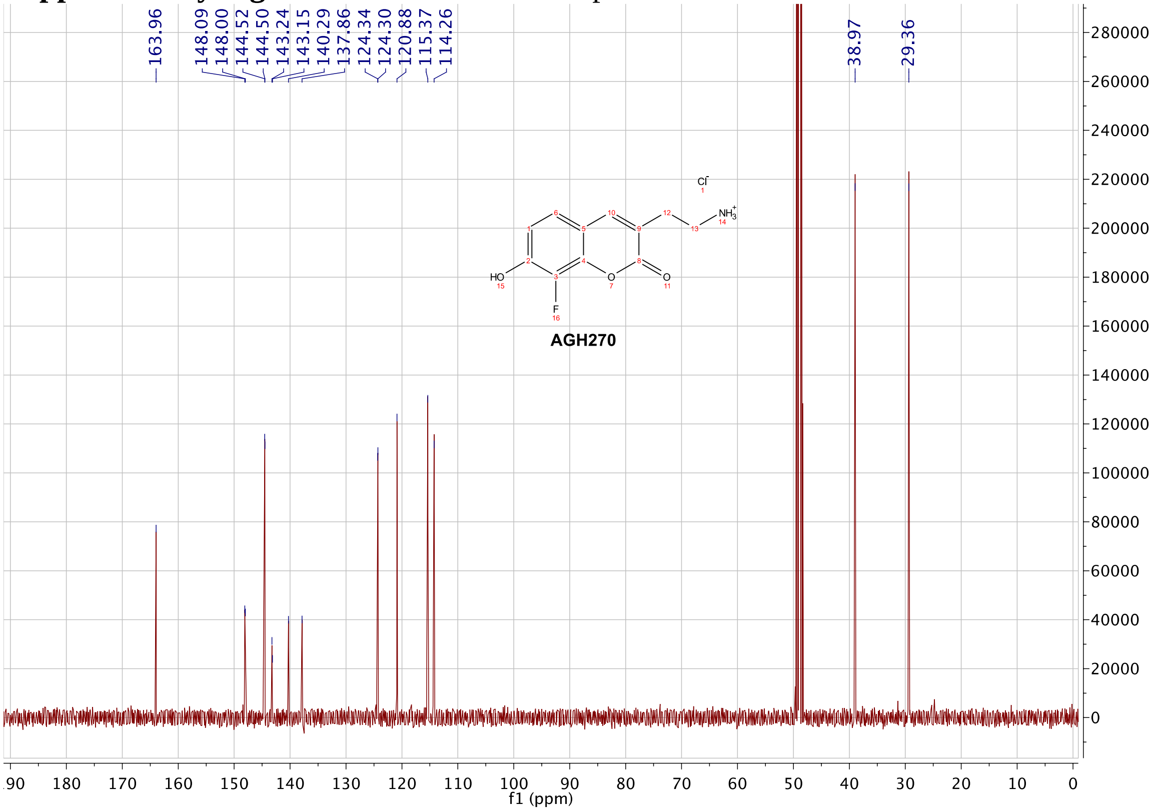

Supplementary Figure 40. ^{19}F-NMR of compound **FFN270**

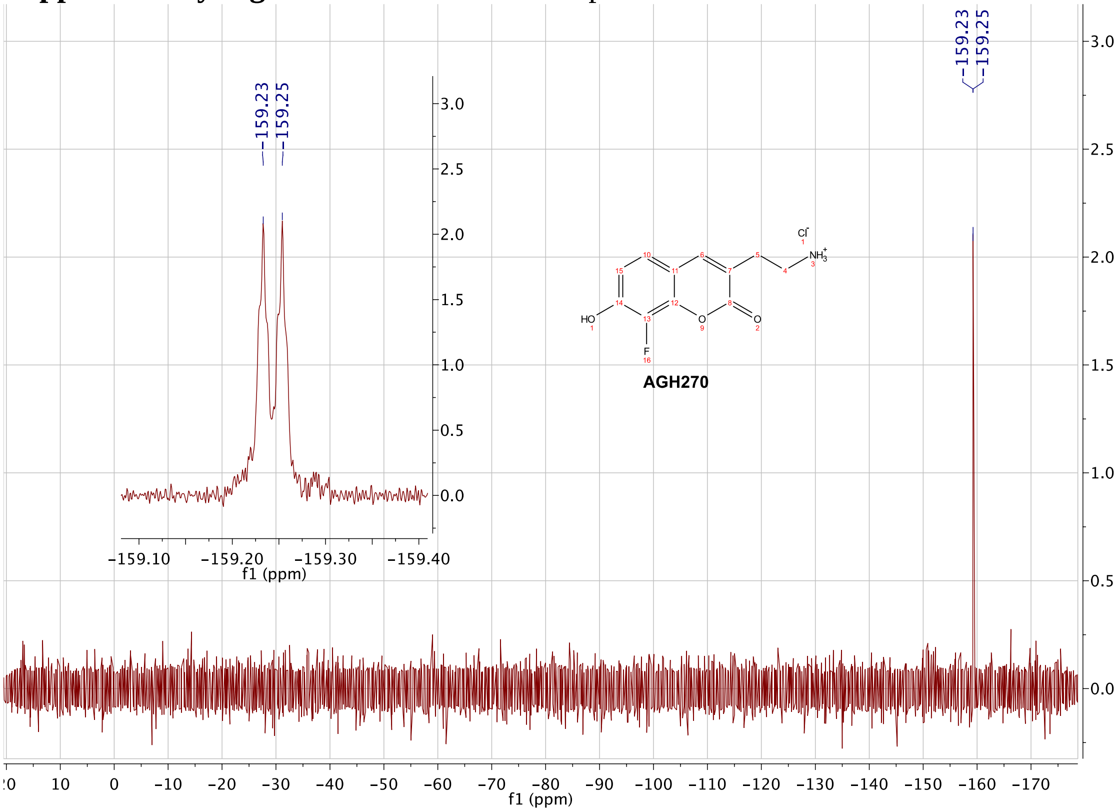

Supplementary References.

1. Besnard, J. *et al.* Automated design of ligands to polypharmacological profiles. *Nature* **492,** 215–220 (2012).
2. Lein, E. S. *et al.* Genome-wide atlas of gene expression in the adult mouse brain. *Nature* **445,** 168–176 (2007).
3. Lee, M., Gubernator, N. G., Sulzer, D. & Sames, D. Development of pH-responsive fluorescent false neurotransmitters. *J. Am. Chem. Soc.* **132,** 8828–30 (2010).
4. Jara, A. N., Torres, M. A., Rezende, M. C. & Cassels, B. K. Some fluoro and nitro analogues of hallucinogenic amphetamines. *Synth. Commun.* **24,** 417–426 (1994).
5. Radomkit, S. *et al.* Pt (IV)-catalyzed generation and [4+ 2]-cycloaddition reactions of o-quinone methides. *Tetrahedron* **67,** 3904–3914 (2011).
6. Fu, Z. *et al.* Decarboxylative Halogenation and Cyanation of Electron-Deficient

Aryl Carboxylic Acids via Cu Mediator as Well as Electron-Rich Ones through Pd Catalyst under Aerobic Conditions. *J. Org. Chem.* **81,** 2794–2803 (2016).

7. Chan, J., Lu, A. & Bennet, A. J. Turnover Is rate-limited by deglycosylation for micromonospora viridifaciens sialidase-catalyzed hydrolyses: Conformational implications for the Michaelis complex. *J. Am. Chem. Soc.* **133,** 2989–2997 (2011).
8. Tan, J. S. & Ciufolini, M. A. Total synthesis of topopyrones B and D. *Org. Lett.* **8,** 4771–4774 (2006).
9. Lukhtanov, E. A. & Andothers. COMPOUNDS AND METHODS FOR FLUORESCENT LABELING. WO/2003/023357 (2003).
10. Saper, N. I. & Snider, B. B. 2,2,6,6-Tetramethylpiperidine-catalyzed, ortho-selective chlorination of phenols by sulfuryl chloride. *J. Org. Chem.* **79,** 809–813 (2014).
